# Aerosol‐Radiation Interactions in China in Winter: Competing Effects of Reduced Shortwave Radiation and Cloud‐Snowfall‐Albedo Feedbacks Under Rapidly Changing Emissions

**DOI:** 10.1029/2021JD035442

**Published:** 2022-05-04

**Authors:** Jonathan M. Moch, Loretta J. Mickley, Christoph A. Keller, Huisheng Bian, Elizabeth W. Lundgren, Shixian Zhai, Daniel J. Jacob

**Affiliations:** ^1^ John A. Paulson School of Engineering and Applied Sciences Harvard University Cambridge MA USA; ^2^ Department of Earth and Planetary Sciences Harvard University Cambridge MA USA; ^3^ Global Modeling and Assimilation Ofﬁce NASA Goddard Space Flight Center Greenbelt MD USA; ^4^ Universities Space Research Association Columbia MD USA

**Keywords:** aerosols, climate, aerosol‐radiation interactions

## Abstract

Since 2013, Chinese policies have dramatically reduced emissions of particulates and their gas‐phase precursors, but the implications of these reductions for aerosol‐radiation interactions are unknown. Using a global, coupled chemistry‐climate model, we examine how the radiative impacts of Chinese air pollution in the winter months of 2012 and 2013 affect local meteorology and how these changes may, in turn, influence surface concentrations of PM_2.5_, particulate matter with diameter <2.5 μm. We then investigate how decreasing emissions through 2016 and 2017 alter this impact. We find that absorbing aerosols aloft in winter 2012 and 2013 heat the middle‐ and lower troposphere by ∼0.5–1 K, reducing cloud liquid water, snowfall, and snow cover. The subsequent decline in surface albedo appears to counteract the ∼15–20 W m^−2^ decrease in shortwave radiation reaching the surface due to attenuation by aerosols overhead. The net result of this novel cloud‐snowfall‐albedo feedback in winters 2012–2013 is a slight increase in surface temperature of ∼0.5–1 K in some regions and little change elsewhere. The aerosol heating aloft, however, stabilizes the atmosphere and decreases the seasonal mean planetary boundary layer (PBL) height by ∼50 m. In winter 2016 and 2017, the ∼20% decrease in mean PM_2.5_ weakens the cloud‐snowfall‐albedo feedback, though it is still evident in western China, where the feedback again warms the surface by ∼0.5–1 K. Regardless of emissions, we find that aerosol‐radiation interactions enhance mean surface PM_2.5_ pollution by 10%–20% across much of China during all four winters examined, mainly though suppression of PBL heights.

## Key Points

- GEOS‐Chem aerosols are for the first time coupled to radiation in an Earth System Model to examine aerosol‐radiation interactions in China
- Absorbing aerosols aloft may reduce clouds and snowfall, causing more surface absorption of radiation despite aerosol attenuation overhead
- Aerosol‐radiation interactions over China in winter can increase concentrations of particulate matter in surface air by ∼10%–20%

## 1 Introduction

Fine particle pollution (PM_2.5_, particulate matter with diameter less than 2.5 μm) is responsible for over 1–2 million deaths per year in China (Cohen et al., 2017; Vohra et al., 2021). In addition to this significant impact on public health, PM_2.5_ – the fine component of the aerosol – affects climate both directly, by influencing how radiation traverses the atmosphere, and indirectly, through its interactions with clouds (Myhre et al., 2013). Given the high aerosol burden over China, changing PM_2.5_ concentrations can strongly influence anthropogenic radiative forcing, temperature trends, and regional meteorology (Liao et al., 2015; K. Li et al., 2016; Miao et al., 2019). Between 2013 and 2018, China enacted a series of policies aimed at reducing PM_2.5_, and surface observations show a dramatic reduction of annual mean PM_2.5_ across China by ∼30%–50% during this period, resulting in large part from these policies (Zhai et al., 2019; Zhang et al., 2019).

Attribution of the decline in PM_2.5_ to emissions reductions is complicated, however, by the variability in meteorology, which can also influence PM_2.5_ concentrations on interannual, seasonal, and daily timescales (Leung et al., 2018; Zhai et al., 2019), as well as on local and synoptic spatial scales. Additionally, quantifying the effect of meteorology on PM_2.5_ abundance in China is made difficult by the feedback of PM_2.5_ onto regional and local meteorology, so that some meteorological variation may actually be driven by emission changes. Aerosol‐radiation and aerosol‐cloud interactions may thus promote extreme haze events, during which PM_2.5_ concentrations can exceed 200 μg m^−3^ (Ding et al., 2016; Miao et al., 2019). Using a global, coupled chemistry‐climate model, we examine the effect of changing aerosol concentrations on both local and regional climate in China, thereby shedding light on the magnitude of aerosol‐radiation feedbacks in the region and the value of emission reduction policies for PM_2.5_ reductions. We focus here only on aerosol‐radiation interactions. Impacts of aerosols on clouds are considered through the influence of aerosol‐radiation interactions on meteorological variables such as temperature or cloud liquid water, also referred to as semi‐direct effects. Neither the aerosol indirect effect, in which aerosols serve as cloud condensation nuclei, nor the effect of aerosol deposition on snow are included in our study.

By reflecting and absorbing light that otherwise would have reached the Earth's surface, aerosols alter the radiation balance of the atmosphere. Aerosol optical depth (AOD) in China frequently nears or exceeds one, especially during heavy haze events (Che et al., 2014, 2015; Li et al., 2013; Zhang et al., 2020). Such events are usually characterized by a significant presence of absorbing aerosols (Xia et al., 2016; Xie et al., 2015). The high levels of absorbing and scattering aerosols lead to a substantial aerosol direct radiative effect, reducing the downward solar flux at the surface and increasing radiation absorbed within the atmosphere (Li et al., 2017; Xia et al., 2016; Zhong, Zhang, Wang, et al., 2018). For example, during a haze event in Beijing in January 2013, Bi et al. (2014) estimated that about half the top of the atmosphere radiative impact of aerosols was due to heating of the atmosphere rather than reflecting radiation back into space.

Such changes in the radiation balance due to the presence of aerosols may lead to a cascade of meteorological effects, including cooling at the surface and warming at the top of the planetary boundary layer (PBL) or just above it (Huang et al., 2018; Li et al., 2017; Miao et al., 2019). Of chief importance for PM_2.5_ pollution is the potential decrease of planetary boundary layer (PBL) height. By reducing temperatures at the surface and increasing temperatures aloft, scattering and absorbing aerosols can increase the thermal stratification of the planetary boundary layer (PBL), suppressing vertical mixing and thereby reducing PBL height (Ding et al., 2016; Huang et al., 2018; Miao et al., 2019). Greater stratification of the PBL can also weaken surface wind speeds by impeding the vertical flux of horizontal momentum from higher altitudes to the surface (Gao et al., 2015; Jacobson & Kaufman, 2006; Zhong, Zhang, Wang, et al., 2018). Cooler surface temperatures, in turn, can increase relative humidity (RH, Liu et al., 2018; Zhong, Zhang, Dong, et al., 2018). Finally, aerosol‐radiation interactions can suppress precipitation locally by reducing surface evaporation and convection, while at the same time enhancing precipitation downwind of polluted areas (Fan et al., 2015; Li et al., 2017; Liu et al., 2018). Warmer temperatures aloft due to absorbing aerosols can also limit cloud formation and subsequent precipitation (Fan et al., 2015; Liao et al., 2015). Aerosols can also indirectly affect cloud formation by acting as cloud condensation nuclei, which can increase the concentration but limit the size of cloud droplets. These changes enhance cloud albedo but decrease precipitation (Liao et al., 2015; Lohmann & Feichter, 2005). On a synoptic scale, observations and models suggest that aerosols can change large‐scale circulation patterns, such as by weakening of the East Asian monsoonal circulation (Jiang et al., 2017; Z. Li et al., 2016; Liu et al., 2019; Niu et al., 2018; Zhang et al., 2012).

These aerosol‐radiation interactions may lead to meteorological feedbacks that alter PM_2.5_ concentrations. Across China, PM_2.5_ abundance is strongly influenced by temperature, PBL height, wind speed, relative humidity (RH), and precipitation (Leung et al., 2018; Zhai et al., 2019). In general, shallower PBL heights are associated with higher levels of surface PM_2.5_ since the PBL governs the volume of air into which surface emissions can mix (Li et al., 2017; Miao et al., 2019). Low wind speeds are similarly associated with higher PM_2.5_ concentrations in regions dominated by anthropogenic emissions due to the decrease in ventilation (Jacob & Winner, 2009; Ji et al., 2014). Precipitation provides a major sink for PM_2.5_ through wet deposition, and reductions in local rainfall can therefore increase PM_2.5_ (Chen et al., 2018; Leung et al., 2018). relative humidity (RH) in the PBL is generally positively correlated with PM_2.5_ in northern China, probably because higher RH in this region is strongly related to declines in PBL height and to the water content of clouds and aerosol, which in turn is linked to the chemical production of PM_2.5_ (Leung et al., 2018; Liu et al., 2018; Moch et al., 2018; Shao et al., 2019; Zhai et al., 2019). However, in southern China, RH is usually negatively correlated with PM_2.5_, possibly because here RH is more strongly associated with precipitation and the southerly airflow that can ventilate the region. Surface temperatures in China are positively correlated with PM_2.5,_ but this may be partly a result of the relationships between temperature and other meteorological variables such as stagnation (Leung et al., 2018; Zhai et al., 2019), as well as the impact of temperature on PM_2.5_ chemical formation (Zhai et al., 2021). At the regional scale, a weaker East Asian winter monsoon is associated with higher levels of PM_2.5_ in East Asia (Cai et al., 2017; Wang et al., 2019).

Many of the meteorological conditions that promote particulate pollution are the same as those that aerosol‐radiation interactions enhance, and so these interactions may act as a positive feedback for PM_2.5_. For example, the PBL feedback exacerbates levels of PM_2.5_ (Miao et al., 2019; Quan et al., 2014; Zheng et al., 2015) and increased PM_2.5_ may further decrease wind speeds (Gao et al., 2015). Indeed, evidence of aerosol‐radiation interactions driving PM_2.5_ pollution has been inferred from observations for multiple regions in China (e.g., Huang et al., 2018; Zhong et al., 2019). For example, such interactions may have enhanced the effectiveness of the brief dramatic reductions in emissions in Beijing in November 2014, when the Asia‐Pacific Economic Cooperation Conference took place and strict air quality measures were enacted (Gao et al., 2017; Zhou et al., 2019).

Multiple modeling studies have shown that accounting for the direct aerosol‐radiation feedbacks is critical for accurate simulation of both pollution concentrations and meteorological conditions of extreme haze episodes (Gao et al., 2015; Miao et al., 2016; Qiu et al., 2017; H. Wang et al., 2018; X. Wang et al., 2018). In general, these prior studies have examined individual haze events in particular locations using regional models with prescribed boundary conditions (e.g., J. Wang et al., 2014; X. Wang et al., 2018; Qiu et al., 2017). In this way, high spatial resolution over the area of interest is achieved. A typical approach is to perform two simulations, one in which aerosols influence radiation and one in which this aerosol effect is turned off. By comparing these simulations, such studies can then estimate the importance of aerosol‐radiation feedbacks for PM_2.5_ concentrations and meteorology during the individual event.

A key shortcoming of such studies, however, is that such feedbacks may be partially dampened by the meteorological boundary conditions used to set up the model domain. Such boundary conditions also generally preclude the possibility of simulating aerosol‐induced changes in large scale circulation patterns. The studies also typically focus on just a few days or weeks and so may not capture aerosol‐radiation interactions occurring on seasonal timescales (e.g., Gao et al., 2015; H. Wang et al., 2018). In addition, examination of just one model year or season provides limited information on the feedbacks, which may vary depending on background meteorology. On the other hand, studies using Earth System Models (ESMs) to examine the effect of Chinese aerosols on climate have frequently been limited by simple representations of chemistry that may not accurately reflect the magnitude of the aerosol burden over China or the processes behind it. Such studies must also overcome issues of heightened internal meteorological variability through the use of longer simulations or ensembles (e.g., Bartlett et al., 2018; Jiang et al., 2017; Lin et al., 2018; Liu et al., 2019).

Our work builds upon previous studies by applying an ensemble of global model simulations to investigate the effect of emissions reductions in China on aerosol‐radiation interactions for winters in 4 years – 2012, 2013, 2016, and 2017. We focus on winter because it has the highest PM_2.5_ concentrations among other seasons in China, and therefore the largest relative impacts on regional climate and meteorology. Using an Earth System Model (ESM) with two‐way coupling to the GEOS‐Chem atmospheric chemistry model, we shed light on the role of aerosol‐radiation feedbacks in influencing observed trends of PM_2.5_ in China. Previous work has coupled GEOS‐Chem to the regional Weather Forecasting Model (WRF) for study of aerosol‐meteorology coupling (Feng et al., 2021), but our work represents the first time that GEOS‐Chem aerosols have been fully coupled with the radiation scheme in an Earth System Model (ESM). The heavy but rapidly changing aerosol burden over China provides a good case study for examining the importance of aerosol‐radiation feedbacks using this new model setup. We focus here only on aerosol‐radiation interactions, and do not consider aerosol indirect effects involving cloud condensation nuclei. By quantifying the possible enhanced effectiveness of emissions reductions due to aerosol‐radiation feedbacks, our work provides guidance for designing and evaluating future air quality policies in China or elsewhere.

## 2 Methods

To examine aerosol‐radiation interactions in China, we couple the aerosol emissions, chemistry, and deposition schemes from GEOS‐Chem with the radiation and transport schemes in Goddard Earth Observing System ESM (GEOS‐ESM). In this section we describe the components of this coupled model (Table 1) and the simulations applied (Table 2). The observations and reanalysis products used for model validation are described in the supplement.

**Table 1:** Summary of Utilized Models

Model name	Description
GEOS‐ESM	Earth System Model developed at NASA Goddard Space Flight Center (Section 2.2, Section S1 in Supporting Information S1)
GOCART	Default aerosol model for GEOS‐ESM. (Section S1 in Supporting Information S1)
GEOS‐Chem	Atmospheric chemistry model used as offline chemical transport model and coupled to GEOS‐ESM (Section 2.2)
GEOS‐GC	GEOS‐ESM fully coupled to GEOS‐Chem, with GEOS‐Chem aerosols affecting radiation (Section 2.3)

*Note*. ESM, Earth System Model; GOCART, Goddard Chemistry, Aerosol, Radiation, and Transport model.

**Table 2:** List of Simulations

Simulation name	Description
GEOS‐GC	Simulation using GEOS‐GC, with GEOS‐Chem aerosols linked to the radiation scheme
GEOS‐GC‐China0	Same as GEOS‐GC, but with aerosols over China transparent to radiation
GC‐Offline	Simulation using GEOS‐Chem as an offline chemical transport model but using the same emissions and chemistry as the other simulations

### 2.1 GEOS‐ESM

The Goddard Earth Observing System ESM (GEOS‐ESM) is developed by the Global Modeling and Assimilation Office at the NASA Goddard Space Flight Center. Goddard Earth Observing System ESM (GEOS‐ESM) is used for forecasts and reanalysis products such as GEOS‐S2S‐2 and MERRA‐2 (Gelaro et al., 2017; Molod et al., 2020). GEOS‐ESM has also been used for longer term prediction (e.g., decadal time scales) and in model intercomparison projects such as CMIP5 and CCMI (Ham et al., 2014; Morgenstern et al., 2017). We briefly describe key aspects of the model in the supplement (S1). Detailed documentation of GEOS‐ESM can be found elsewhere (e.g., Molod et al., 2015, 2012; Nielsen et al., 2017; Rienecker et al., 2008).

The Goddard Chemistry, Aerosol, Radiation, and Transport model (GOCART) is the default aerosol chemistry module online in GEOS‐ESM (Chin et al., 2000, 2002; Colarco et al., 2010), providing aerosol mass to the radiation code, which then uses the mass concentrations to calculate aerosol optical depth and the impacts on absorption and scattering of shortwave and longwave radiation throughout the atmosphere. More details on Goddard Chemistry, Aerosol, Radiation, and Transport model (GOCART) are in the supplement (S1).

### 2.2 GEOS‐Chem

GEOS‐Chem is a grid‐independent global/regional atmospheric chemistry model including a detailed aerosol‐oxidant chemical mechanism coupled to emissions, deposition and transport (Alexander et al., 2012; Park et al., 2004; Pye et al., 2009; Sherwen et al., 2016; Travis et al., 2016). The model includes a stand‐alone, grid‐independent chemical module applying local operations (chemistry, emissions, deposition) on individual atmospheric columns, coupled to transport modules applying advection, convection, and boundary layer turbulence (Long et al., 2015). The standard application of GEOS‐Chem is as an off‐line chemical transport model (CTM) using archived meteorological data from GEOS‐ESM analyses. However, the stand‐alone chemical module can also be coupled to a meteorological model for on‐line simulations in which the meteorological model handles the chemical transport. GEOS‐Chem has been coupled in this manner to the GEOS‐ESM (Hu et al., 2018), to the Beijing Climate Center ESM (Lu et al., 2020), and to Weather Forecasting Model (WRF) (Feng et al., 2021; Lin et al., 2020).

GEOS‐Chem as a chemical transport model (CTM) has been used for numerous studies examining PM_2.5_ and its components over China including evaluation with observations and analysis of aerosol chemistry (e.g., Dang & Liao, 2019; Shao et al., 2019; Zhai et al., 2021). The more complex chemical mechanism in GEOS‐Chem allows for a better representation of aerosol processes over China than that provided by Goddard Chemistry, Aerosol, Radiation, and Transport model (GOCART), which uses prescribed fields for many chemically active gas‐phase species. In model comparisons with observed satellite Aerosol optical depth (AOD) over China, GEOS‐Chem tends to outperform GOCART (e.g., Cheng et al., 2012; S. Li et al., 2016).

The model as used here (version 12.7.0, https://doi.org/10.5281/zenodo.3634864) includes bulk representations of sulfate‐nitrate‐ammonium (SNA) aerosol, organic aerosol (OA), and black carbon (BC), dust in four size classes, and sea‐salt aerosol in two size classes. Sulfate and nitrate chemistry are as described by Alexander et al. (2012) and Shah et al. (2020), and sulfate‐nitrate‐ammonium (SNA) thermodynamics follow ISORROPIA II (Fountoukis & Nenes, 2007). Sulfate and nitrate are also produced in sea‐salt aerosol (Alexander et al., 2005). black carbon (BC) and organic aerosol (OA) in GEOS‐Chem are divided into hydrophilic and hydrophobic fractions (Pye et al., 2009; Q. Wang et al., 2014). In addition to primary organic aerosol (OA), we use a simple scheme for secondary organic aerosol (SOA), which applies a fixed secondary organic aerosol (SOA) yield from reactions of precursor gases (Pai et al., 2020). Aerosols are removed by wet and dry deposition following Liu et al. (2001) and Zhang et al. (2001), with additional formulations for black carbon (BC) scavenging (Q. Wang et al., 2014), wet deposition updates by Luo et al. (2019), and deposition of HNO_3_ to snow (Jaegle et al., 2018). Photolysis rates in GEOS‐Chem are calculated using the FAST‐JX photolysis scheme, which takes into account the effects of aerosols on radiative flux (Bian & Prather, 2002; Eastham et al., 2014; Mao et al., 2010). As GEOS‐Chem uses a bulk aerosol scheme, heterogeneous reactions on aerosols are parameterized as pseudo‐first order reactions (e.g., McDuffie et al., 2018). Here we have modified version 12.7.0 to include hydroxymethanesulfonate (HMS) aerosol chemistry and revised the cloud water pH calculation for in‐cloud processes (Moch et al., 2018, 2020).

Global anthropogenic emissions are from the Hemispheric Transport of Air Pollution (HTAP; Janssens‐Maenhout et al., 2015) inventory for 2010. Over China, the Hemispheric Transport of Air Pollution (HTAP) emissions are overwritten by the Multi‐resolution Emission Inventory for China (MEIC; Zheng et al., 2018) for 2012–2017, which takes into account recent Chinese government interventions to reduce emissions. Open fire emissions are from the Global Fire Emissions Data set (GFED; van der Werf et al., 2017), and biogenic volatile organic compound emissions follow the Model of Emissions of Gases and Aerosols from Nature (MEGAN, Guenther et al., 2012). Dust and sea salt aerosols emissions are described as functions of wind speed and particle size (Fairlie et al., 2010; Jaegle et al., 2011). Anthropogenic dust is added to the smallest dust size bin (i.e., diameter less than 1 μm), following Philip et al. (2017).

### 2.3 Coupling GEOS‐Chem and GEOS‐ESM

Previous implementation of GEOS‐Chem within GEOS‐ESM has relied on one‐way coupling, in which chemical species are transported by the meteorological model but do not influence meteorology (Hu et al., 2018; Keller et al., 2021; Long et al., 2015). Monthly average aerosol fields from GEOS‐Chem have also been used to influence climate and meteorology in the GISS Global Climate Models (GCM), but with changes in the GISS meteorology having no impact on GEOS‐Chem (Leibensperger et al., 2012a, 2012b). Here we expand on this work by implementing two‐way coupling of GEOS‐Chem with GEOS‐ESM via connection of GEOS‐Chem aerosols to the GEOS‐ESM radiation scheme. This setup allows examination of rapid interactions between chemistry and dynamics, such as that occurring during pollution episodes in China. Such interactions are dampened when using GOCART aerosols, as the use of prescribed fields for multiple gas‐phase species limits the response of chemistry to changes in dynamics. At every model time step in GEOS‐ESM, we apply boundary layer mixing and update the GEOS‐Chem emissions and deposition before calculating chemical production and loss processes. In this way, we avoid excess removal of pollutants via dry deposition (Hu et al., 2018). The GEOS‐ESM convection module does not account for chemical scavenging by precipitation, and therefore we use the GEOS‐Chem convection scheme (Wu et al., 2007) driven by local GEOS‐ESM mass fluxes (Hu et al., 2018). Using the GEOS‐Chem convection scheme does not introduce significant errors in transport relative to using the GEOS‐ESM scheme (Yu et al., 2018).

The connection of GEOS‐Chem aerosols to the radiation code in GEOS‐ESM is done by replacing the default GOCART aerosol mass with GEOS‐Chem aerosol mass. Aerosol optical properties in GEOS‐ESM are not altered. The method of mass replacement varies by aerosol species. For BC, OA, and ammonium, we simply overwrite the GOCART aerosol mass with that from GEOS‐Chem. We also overwrite GOCART sulfate with the sum of sulfate plus hydroxymethanesulfonate (HMS) from GEOS‐Chem. We treat hydroxymethanesulfonate (HMS) as having the same molecular weight as sulfate, which approximates how HMS may be misinterpreted in observations (Moch et al., 2020). For dust and sea salt aerosol, we partition aerosol mass by size. The four smallest size bins for dust have the same bounds in GEOS‐Chem and GOCART, so we simply replace the GOCART aerosol mass in each bin with that from GEOS‐Chem. GOCART has an additional fifth size bin, which represents dust aerosol with particle radii >6 μm. Given that aerosol of this size settles quickly and has minimal effect on radiation, we set the mass in this size bin to zero for this study. For sea salt aerosol, we remap the sea salt aerosol mass concentrations from the two size bins in GEOS‐Chem onto the GOCART size bins, assuming that GEOS‐Chem sea salt aerosol is lognormally distributed with a fine and coarse mode and thus preserving total sea salt aerosol mass (Kodros & Pierce, 2017). Finally, GEOS‐Chem treats sea salt nitrate separately from other forms of nitrate, while GOCART partitions all nitrate into three size bins. We first replace the smallest nitrate size bin in GOCART with non‐sea salt nitrate from GEOS‐Chem. We next partition the GEOS‐Chem sea salt nitrate into coarse and fine fractions based on the ratio of coarse‐to‐fine sea salt in each grid box. The fine and coarse fraction of sea salt nitrate of GEOS‐Chem is then remapped onto the three size bins of GOCART nitrate.

### 2.4 Simulations Overview

Each simulation we conduct employs a c90cubed sphere (∼1° x 1°) horizontal resolution, with 72 layers in the vertical. Two main types of simulations are performed. For the first type, GEOS‐Chem aerosols are linked to the radiation scheme, allowing online feedbacks between aerosols and meteorology (GEOS‐GC). For the second type, we again connect GEOS‐Chem aerosols to GEOS‐ESM radiation, but make the aerosols over China transparent to solar radiation (GEOS‐GC‐China0). To accomplish this, we multiply the aerosol mass sent to the radiation code by a mask, built on a global 1° × 1° grid that zeros out aerosol mass within the geographic boundaries of China and applies a gradient in aerosol mass moving outward from these boundaries (Figure S1 in Supporting Information S1). For those grid boxes at the border adjacent to the zeroed‐out boxes, we assign a value of 0.1. For each grid box adjacent to a 0.1 grid box and not already given a value, we assign a value of 0.2. We repeat this procedure outward from the borders until we reach a value of 1.0. All other grid boxes across the globe are assigned a value of 1.0. We apply this mask to all aerosol species except for dust and sea‐salt, as most dust and all sea‐salt are derived from natural sources. Setting up the mask in this way allows us to avoid a sharp gradient in aerosol mass, which would affect meteorology in ways different from what might be expected from gradual removal of anthropogenic emissions. The mask does not directly affect the calculation of photolysis rates in GEOS‐Chem, but photolysis rates can differ between GEOS‐GC and GEOS‐GC‐China0 insofar as changes to radiation may impact cloud cover.

By comparing these two types of simulations, GEOS‐GC and GEOS‐GC‐China0, we can assess the role of aerosol‐radiation interactions driven mainly by pollution within China. In GEOS‐GC, two‐way coupling between aerosols and radiation is allowed, while in GEOS‐GC‐China0 the meteorology influences PM_2.5_ concentrations but aerosols over China do not affect radiation. Conducting simulations with anthropogenic emissions over China turned off would offer an alternative way to capture the effect of aerosols on regional climate and meteorology (e.g., Jiang et al., 2017; Y. Wang et al., 2014), but this approach would not allow us to quantify the subsequent feedback of meteorology onto air quality. Another possible approach would be to zero out globally all aerosol mass sent to the radiation scheme. This approach has the benefit of being globally consistent, but a major drawback would be that it could highlight the impact of non‐Chinese aerosols on large‐scale circulation patterns, which in turn could influence air quality in China. For example, we found previously that aerosols over Russia can perturb the Siberian High, a driver of weather and air quality in China (Moch, 2020). We therefore settle on the gradient masking approach as the one that most closely captures the effects of aerosol‐radiation interactions on PM_2.5_ concentrations within China.

To examine the effect of changing emissions on aerosol‐radiation feedbacks over China, we perform each simulation type for two 2‐year periods, 2012–2013 and 2016–2017. Winter of 2012–2013 or 2016–2017 refers to the six winter months across the two year period, starting with January of 2012 or 2016. Observed PM_2.5_ decreased by 30%–40% across much of China between 2013 and 2017 in response to decreases in emissions (Zhai et al., 2019; Zhang et al., 2019). These two time windows can thus provide information on whether the magnitude and sign of the feedbacks change as emissions and surface PM_2.5_ concentrations change. For each time period we prescribe the same sea surface temperatures (SSTs) as those used for MERRA‐2 assimilated meteorology (Gelaro et al., 2017).

Regional models examining the impacts of aerosol‐radiation interactions generally use only a single simulation. This is acceptable as the signal is large, the geographic region analyzed is small, and meteorological boundary conditions can dampen internal model variability (e.g., H. Wang et al., 2018; X. Wang et al., 2018). Earth System Models diagnosing global responses to changes in aerosols, on the other hand, often require many more data points to diagnose a clear global signal due to internal model variability, although some regional responses can be inferred quickly if sea surface temperatures are fixed (Forster et al., 2016). Given the large aerosol burden over China, Global Climate Models (GCM) using prescribed sea surface temperatures (SSTs) can diagnose the impacts of Chinese emissions on regional climate and meteorology with relatively few model months. For example, the impact of anthropogenic Asian emissions on Pacific storm tracks was detected with just 12 winter months simulated by a Global Climate Models (GCM) using fixed sea surface temperatures (SSTs) (Y. Wang et al., 2014) and the impact of such emissions on the East Asian Winter Monsoon was diagnosed with 30 winter months using a similar GCM simulation (Jiang et al., 2017).

For each simulation type and each time period, we perform 5‐member ensemble simulations to account for internal model variability in aerosol‐radiation feedbacks. This results in a total of 30 winter months for each period. To initialize each simulation, we start with January 1 conditions from a 20‐year freely running GEOS‐ESM simulation with c90 horizontal resolution and 1992–2011 specified SSTs. A 1‐year spin‐up of the model with GEOS‐Chem online is carried out as follows. We first simulate 11 months in freely running mode, using emissions and specified SSTs from 2011 for the first time period and from 2015 for the second time period. The spin‐up allows the longer‐lived species in GEOS‐Chem to adjust to the appropriate meteorology. In the final month of spin‐up with GEOS‐Chem, the model is nudged to MERRA‐2 assimilated meteorology in order to set the atmosphere to a realistic state (Orbe et al., 2017). Each of the five ensemble members is nudged by MERRA‐2 meteorology from a different year, thus generating five different sets of initial conditions for the subsequent 2‐year simulation periods. We return the model to freely running mode for the final 2 years, thus allowing two‐way interaction between the meteorology and aerosols to take place. We apply 2012–2013 emissions and SSTs for the first time period and 2016–2017 emissions and SSTs for the second time period. Differences between the two ensembles of simulations, while influenced in part by the SSTs applied, will also reveal how aerosol‐radiation feedbacks over China have changed in response to the steep decline in emissions.

To test for statistical significance of our findings, we conduct either paired *t*‐tests on the differences between the GEOS‐GC and GEOS‐GC‐China0 monthly means in each grid box or pooled *t*‐tests for the simulation monthly mean differences for each ensemble member across the two time periods. For paired *t*‐tests, we pair the simulations by the year used to generate initial conditions. We also account for serial autocorrelation within each ensemble member, which could bias tests for statistical significance (Mickley et al., 2012). For the threshold of statistical significance, we use a p‐value of less than 0.05. Grid boxes that do not meet this threshold are presented as having zero difference.

Finally, to characterize possible biases in meteorology arising in the freely running GEOS‐GC simulations, we compare the GEOS‐GC meteorological fields with MERRA‐2 (Figure S2 in Supporting Information S1). To further assess the impact of meteorological model biases on chemistry, we compare our results with those from the GEOS‐Chem chemical transport model (GC‐offline) driven by MERRA‐2 assimilated meteorology (Figure S3 in Supporting Information S1, Zhai et al., 2021).

## 3 Results

We first show simulated wintertime PM_2.5_ concentrations for the two time periods. We next describe the effect of aerosol‐radiation interactions on meteorology and surface PM_2.5_ for winter 2012–2013. We then examine how changing emissions between 2012–2013 and 2016–2017 alter the impact of aerosol‐radiation interactions between these two periods. Comparisons of GEOS‐GC with GC‐Offline and with MERRA‐2 are included in the Supporting Information S1 (Section S2, Figures S2 and S3).

### 3.1 Simulated PM_2.5_ Concentrations in Winters 2012–2013 and 2016‐2017

**Figure 1:**
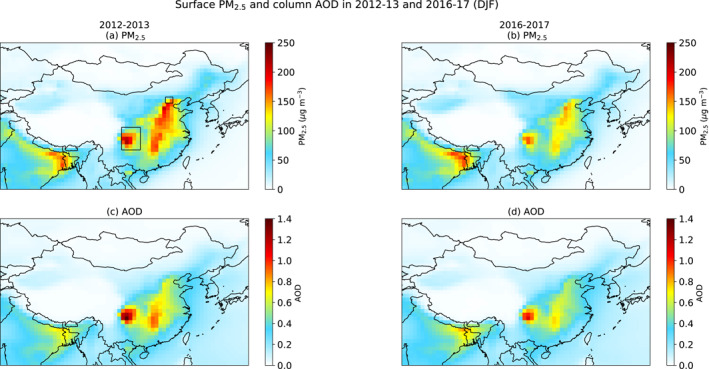
Simulated surface PM_2.5_ concentrations for December‐January‐February (DJF) in GEOS‐GC for (a) 2012–2013 and (b) 2016–2017 and column Aerosol optical depth (AOD) at 550 nm for (c) 2012–2013 and (d) 2016–2017. In the top left panel the larger box over central China shows the location of the Sichuan Basin while the smaller box in northeastern China shows the location of Beijing municipal region.

For winter 2012–2013, GEOS‐GC simulates mean winter surface PM_2.5_ concentrations of ∼150–200 μg m^−3^ for much of eastern China (Figure 1a). In this first time period, PM_2.5_ concentrations show a local maximum over the Sichuan Basin, indicated by the larger box in Figure 1a, where mean PM_2.5_ levels approach 250 μg m^−3^. Simulated PM_2.5_ for the Beijing region, indicated by the smaller box, ranges from ∼125 to 175 μg m^−3^. We find that surface PM_2.5_ concentrations simulated by GEOS‐GC decrease by ∼20–40 μg m^−3^ (∼20%) across eastern China between 2013–12 and 2016–2017 as emissions decline (Figure 1), a trend similar in magnitude to the observed decrease of ∼10–50 μg m^−3^ in surface PM_2.5_ in this region between 2013 and 2018 (Zhai et al., 2019). Simulated PM_2.5_ across much of eastern China in winter 2016–2017 thus averages ∼125 μg m^−3^, with PM_2.5_ concentrations approaching ∼175 μg m^−3^ in the Sichuan Basin (Figure 1b).

### 3.2 Effect of Aerosol‐Radiation Interactions on Meteorology and Surface PM_2.5_ in Winter 2012–2013

The net impact of aerosol‐radiation interactions on PM_2.5_ concentrations and meteorology is a combination of changes across different spatial scales. We first examine local changes and then focus on regional‐scale changes in circulation patterns during winter 2012–2013. Under all‐sky (cloudy and cloud free) conditions, we find that anthropogenic aerosols over China reduce shortwave radiation reaching the surface (i.e., the downward radiative flux at the surface) by 10–15 Wm^−2^ for most of eastern China; a similar amount of shortwave radiation is absorbed within the atmospheric column (Figures S4a and S4c in Supporting Information S1). Over north‐central and northwestern China, aerosols absorb ∼5 W m^−2^ of shortwave radiation within the column (Figure S4c in Supporting Information S1). Under clear‐sky (i.e., cloud‐free) conditions, the impact of aerosol on shortwave radiation reaching the surface nearly doubles while the impact of aerosols on absorption increases slightly (Figure S4b and S4d in Supporting Information S1). The remainder of this paper focuses on results under all‐sky conditions.

**Figure 2:**
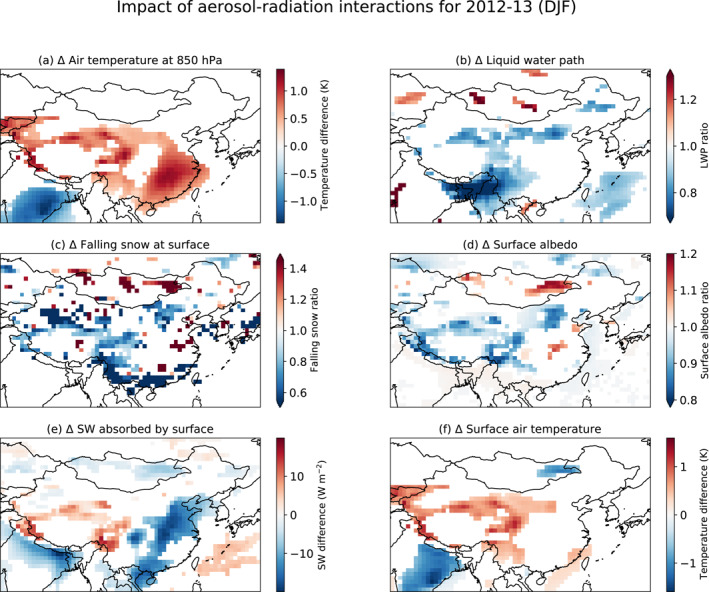
Feedbacks involving aerosol, clouds, snow, and surface albedo. Panels show the impacts of aerosol‐radiation interactions during 2012–2013 winter months (DJF) over China on (a) air temperature at 850 hPa, (b) liquid water path (LWP), (c) falling snow at the surface, (d) surface albedo, (e) shortwave radiation (SW) absorbed by the surface, and (f) surface air temperature. Impacts are shown as the difference (a, e, f) or ratio (b, c, d) between the ensemble mean for GEOS‐GC and of GEOS‐GC‐China0. Colored areas indicate those regions where differences are statistically significant (*p* < 0.05).

The absorption by aerosols in the shortwave leads to substantial heating of the lower troposphere (Figure S4e in Supporting Information S1), with an increase in air temperature at 850 hPa of ∼0.5–1 K over much of southeastern China, central China, and around the edges of the Tibetan Plateau in winter 2012–2013 (Figure 2a, Figure S5 in Supporting Information S1). Higher temperatures, in turn, make condensation of cloud liquid water less likely, and the cloud liquid water path is correspondingly reduced near the eastern edge of the Tibetan Plateau and across northern China at ∼40°N (Figure 2b). In contrast, cloud liquid water remains roughly constant in southeastern China, despite the increase in air temperature. The lack of response in cloud liquid water in southeastern China can be traced to relatively high baseline levels of moisture and temperature, which make increases in air temperature less impactful on the already favorable conditions for cloud formation and precipitation (Figures S6a–S6d in Supporting Information S1). The larger response in cooler and drier regions such around Tibetan Plateau is the same effect in the opposite direction, where small changes in heating rate can lead to more significant changes in cloud cover.

We find that aerosol‐radiation interactions decrease snowfall at the surface by ∼20% near the edges of the Tibetan Plateau and in northeastern and northwestern China in winter 2012–2013. The spatial pattern of changes in snowfall roughly aligns with the pattern of decreasing cloud liquid water path, with declines seen in Central China along the Eastern Boundary of the Tibetan Plateau and across Northern China along the northern boundary of the Tibetan Plateau and extending toward Beijing (Figure 2c). Changes in falling rain in this region are minimal (not shown), indicating that snowfall changes are due primarily to a decrease in total precipitation rather than melting of falling snow. Snowfall also decreases in southern China, but the small amount of snow in this region makes this change minimal in absolute terms (Figure S6e and S6f in Supporting Information S1). Snow cover is an important factor in surface albedo in winter, and we find that the reduction in snowfall leads to a decrease in surface albedo of ∼5%–10% in areas around the Tibetan Plateau and in northeastern China (Figure 2d). In western China, the loss of snow cover increases absorption of shortwave radiation at the surface by ∼5–10 Wm^−2^ (Figure 2e). In most of eastern China, however, shortwave radiation absorbed at the surface decreases by ∼10–15 Wm^−2^, driven largely by the attenuation of shortwave radiation by aerosols aloft (Figure S4a in Supporting Information S1). For north‐central and parts of northeastern China, absorbed shortwave radiation at the surface changes little, as the effects of decreasing albedo and increasing aerosol reflection and absorption aloft appear to balance out.

Aerosol‐radiation interactions in winter 2012–2013 increase surface air temperatures in areas bordering the Tibetan Plateau by ∼0.5–1 K (Figure 2f). The temperature change is due in large part to the increase in shortwave radiation absorbed at the surface with less snow cover present (Figure 2e). Prevailing westerly winds at the surface and in the lower troposphere carry this relatively warmer air eastward across north‐central China (Figure S7a and S7b in Supporting Information S1), counteracting the surface cooling that would otherwise occur due to the aerosol burden aloft.

**Figure 3:**
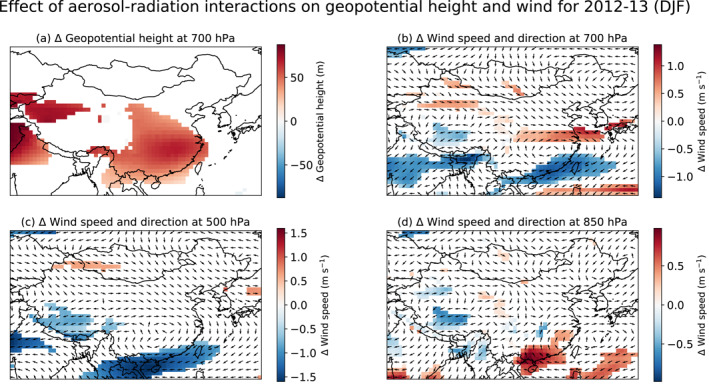
Impact of aerosol‐radiation interactions on atmospheric circulation patterns for 2012–2013 in December‐January‐February (DJF). The panels show the changes in (a) geopotential height and in wind speed and direction at (b) 700 hPa, (c) 500 hPa, and (c) 850 hPa. The impacts are calculated as the difference between the ensemble mean for GEOS‐GC and GEOS‐GC‐China0. Only statistically significant changes (*p* < 0.05) are shown for geopotential height and wind speed. Arrows for (b–c) show the net direction of the wind changes. Blue‐shaded regions thus indicate a decrease in wind speed in the opposite direction of the overlying arrows.

GEOS‐GC yields a large aerosol burden over the Sichuan Basin, with mean wintertime Aerosol optical depth (AOD) as much as 1.4 (Figure S1 in Supporting Information S1). We find that the aerosol perturbs regional circulation patterns and affects air temperatures both aloft and at the surface. In the middle‐ to lower troposphere, regional circulation is dominated by the East Asian winter monsoon (Z. Li et al., 2016), with strong northwesterly winds flowing from the semi‐permanent Siberian High and northerly surface winds in eastern China and along the coast (Figure S7 in Supporting Information S1). In winter 2012–2013, the aerosols over the Sichuan Basin induce a shortwave heating of ∼0.3–0.5 K day^−1^ at 700 hPa (Figure S9a in Supporting Information S1). Prevailing wind patterns, however, complicate the relationship between the changes in shortwave heating due to aerosols and the changes in temperature. The East Asian Jet, part of the East Asian monsoon circulation, carries this warm air eastward (Figure S7 in Supporting Information S1), causing an increase in air temperature of ∼0.5 K at 700 hPa about 1,000 km east of the Basin (Figure 2a). These warmer temperatures lead to an increase in geopotential height of ∼70 m at 700 hPa (Figure 3a). Given that geopotential heights over China decrease poleward, this perturbation increases the gradient in geopotential heights just north of ∼29°N over eastern China and decreases the gradient south of that, thereby speeding up the East Asian Jet in the north by ∼0.5–1 m s^−1^ at 700 hPa but slowing the Jet by ∼1 m s^−1^ in the south (Figure 3b). The slowdown in the south, in turn, exerts drag on winds at lower altitudes, facilitating the flow of warm, marine air into southern China at 850 hPa (Figure 3d) and limiting outflow of pollution at the surface (Figure S7 in Supporting Information S1). This weakening of the East Asian monsoon leads to a slight warming of ∼0.5 K off the southern coast of China (Figure 2f).

**Figure 4:**
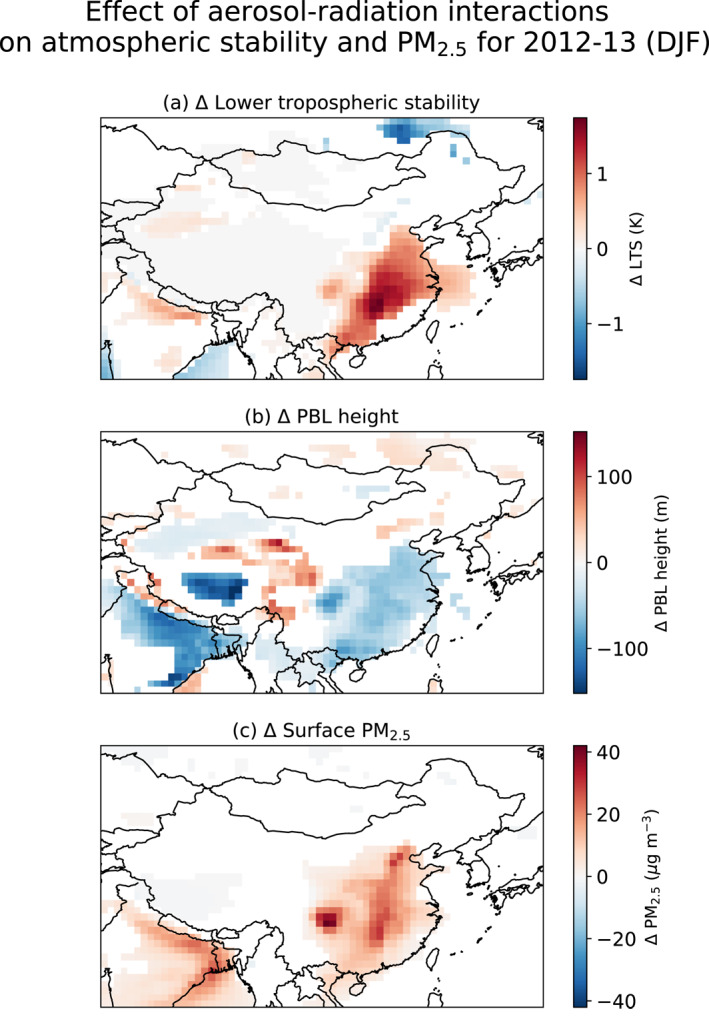
Impact of aerosol‐radiation interactions on atmospheric stability and surface PM_2.5_ concentrations. Panel (a) shows the change in lower tropospheric stability, defined as the difference in potential temperature between 850 and 1,000 hPa, due to aerosol‐radiation interactions; panel (b), the change in planetary boundary layer height; and panel (c), the change in surface PM_2.5_. The impacts are shown as difference between the ensemble mean for GEOS‐GC and GEOS‐GC‐China0. Colored areas indicate those regions where differences are statistically significant changes (*p* < 0.05).

Despite the absence of a significant surface temperature response to aerosol‐radiation interactions for much of eastern China, the substantial warming of the mid‐troposphere in winter 2012–2013 nevertheless steepens the vertical potential temperature gradient in the lower troposphere over most of the region, thus enhancing atmospheric stability. Figure 4a shows the effect of aerosol‐radiation interactions on lower tropospheric stability (LTS), defined here as the difference in potential temperature at 850 and 1,000 hPa (Wood & Bretherton, 2006). We find that LTS increases by ∼1 K (10%–20%) for most of eastern China south of ∼39°N. The increase in LTS reflects a suppression of vertical mixing and decreases PBL heights by ∼50 m (∼10%) (Figure 4b). Along the eastern and northern edges of the Tibetan Plateau, the surface warming due to the proposed cloud‐snowfall‐albedo feedback dominates the atmospheric response, resulting in little change in LTS but an increase in PBL height of ∼50–100 m. Across the central Tibetan Plateau, a slowdown in wind speeds in the lower troposphere (Figures 3b and 3d) reduces overall turbulence and thus PBL height by ∼140 m.

By decreasing PBL height and enhancing stability, we find that aerosol‐radiation interactions over China generally limit the volume of air into which surface PM_2.5_ mixes and reduce the vertical dispersion of pollutants. In addition, the aerosol‐induced weakening of the East Asian monsoon in southern China reduces ventilation of pollutants. Taken together, these effects lead to an increase in surface PM_2.5_ of ∼10–40 μg m^−3^ (∼10%–20%) across eastern China in winter 2012–2013 (Figure 4c). The Sichuan Basin, the region with the highest simulated pollution, shows the largest absolute increase in surface PM_2.5_, ∼40 μg m^−3^. Despite changes in PBL height over the Tibetan Plateau, there is little PM_2.5_ in this region, and so the perturbation in surface PM_2.5_ for this region is minimal.

### 3.3 Effect of Changing Emissions on Aerosol‐Radiation Interactions and Associated Impacts

The widespread decrease in PM_2.5_ concentrations between the two periods (2012–2013 vs. 2016–2017) results in a decline of ∼3 W m^−2^ (∼5%) in shortwave radiation absorbed by aerosols aloft over eastern China (Figure S11a in Supporting Information S1). The change in shortwave radiation absorption reduces shortwave heating by ∼0.1 K day^−1^ (∼5%–10%) at 850 hPa over eastern China, excluding the southeast (Figure S11b in Supporting Information S1). As PM_2.5_ decreases, the single scattering albedo, averaged through the column, also slightly increases for northeastern China (Figure S12 in Supporting Information S1). The decline in shortwave heating of the lower troposphere due to aerosols in 2016–2017 leads to less cloud evaporation and thus increases snowfall and surface albedo in parts of north China, compared to 2012–2013 (Figure S11c–S11e in Supporting Information S1). The distributions of cloud liquid water path (LWP) are extremely similar between the two periods, except for a slight decline in cloudiness in Southeastern China (Figure S6c and S17c in Supporting Information S1). This result suggests that, outside of Southeastern China, the differing response of cloud liquid water path (LWP) to aerosols in the two periods (Figure 2b and Figure S13b in Supporting Information S1) is mostly due to changes in aerosol concentrations rather than baseline clouds. The baseline temperature spatial pattern at 850 hPa is also very similar between the two time periods (Figure S18 in Supporting Information S1).

In addition to influencing the cloud‐snowfall‐albedo feedback, declines in aerosols also reduce reflected shortwave radiation, which competes with the impacts of surface albedo changes. The relative importance of the reflection due to aerosols versus the cloud‐snowfall‐albedo feedback varies geographically in both time periods. The competition between these two mechanisms can therefore lead to differing spatial patterns across the two periods for the impacts of aerosols on meteorological variables such as temperature (Figure 2 and Figure S13 in Supporting Information S1). As the Figures show only changes that are statistically different from zero, a reduction in the magnitude of the response to aerosols can also appear to change the spatial pattern of the response.

In northwestern China, concentrations of dust in surface air increase by ∼30% in 2016–2017, as discussed below. This increase in dust along with the enhanced surface albedo leads to ∼1 W m^−2^ more atmospheric absorption due to aerosol‐radiation interactions for 2016–2017 compared to 2012–2013 (Figure S11a in Supporting Information S1). The presence of anthropogenic aerosols aloft amplifies dust absorption through scattering of incoming sunlight. The aerosol‐induced increase in surface albedo, compared to induced reduction in 2012–2013, means that more sunlight is reflected back to space, allowing a second pass through the atmosphere. The enhanced absorption increases the aerosol impact on shortwave heating by 0.05 K day^−1^ over northwestern China (Figure S10b in Supporting Information S1).

**Figure 5:**
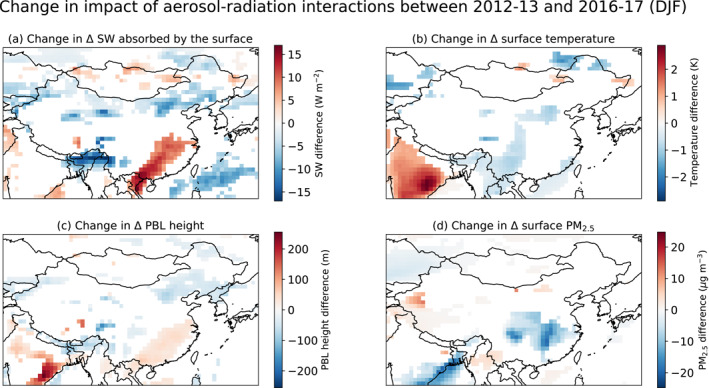
Change in the impacts of aerosol‐radiation interactions from 2012–2013 to 2016–2017 (DJF) for (a) shortwave radiation (SW) absorbed by the surface, (b) surface air temperature, (c) planetary boundary layer (PBL) height, and (d) surface PM_2.5_ concentrations. All changes are shown as the difference between aerosol‐induced effects for 2016–2017 versus those effects for 2012–2013 – that is, the differences between the difference of the ensemble means for GEOS‐GC and GEOS‐GC‐China0 in each of the two time periods. Colored areas indicate those regions where differences are statistically significant (*p* < 0.05).

Across northern China, declines in the proposed cloud‐snowfall‐albedo feedback play a larger role than declines in shortwave reflection by aerosols. In 2016–2017, aerosols thus decrease absorption of shortwave radiation at the surface by ∼5 W m^−2^ (Figure S13e in Supporting Information S1), a 5–10 W m^−2^ reduction relative to 2012–2013 (Figure 5a). The weakening of this feedback leads in turn to cooler surface temperatures of ∼0.5–1 K in northeastern China in 2016–2017 (Figure S13f in Supporting Information S1), ∼0.5 K cooler than the impact in 2012–2013 (Figure 5b). The cooling impact in the northeast also leads to a more stable lower troposphere and ∼50 m lower PBL heights (Figure 5c, Figures S14a and S14b in Supporting Information S1).

In contrast, over southeastern China in 2016–2017, the declines in reflection of shortwave radiation by aerosols, along with changes in clouds, are more important than surface albedo changes. The reduction in aerosol aloft increases shortwave radiation absorbed by the surface relative to 2012–2013 by ∼8–14 W m^−2^ (Figure 5a, Figure S13e in Supporting Information S1). However, transport of cooler air from the north and west counteracts the local effect of greater absorption of shortwave radiation at the surface, as discussed below, leading to little net change in surface temperatures in 2016–2017 (Figure S13f in Supporting Information S1), similar to the impact in the first time period (Figure 5b). The reduced warming attributed to aerosols in the middle‐ and lower troposphere in conjunction with only minor changes in surface air temperatures leads to an overall decrease in PBL stratification and thus a slight increase of ∼50 m in PBL heights (Figure 5c).

Although the baseline circulation patterns are similar in winter 2012–2013 and winter 2016–2017 (Figure S7 and S19 in Supporting Information S1), differences in the response of these patterns to aerosol‐radiation interactions are likely driven by both changing aerosol load as well as the different SSTs applied. We attribute reductions in geopotential heights at 700 hPa by ∼25–75 m relative to those in the first period (Figure S15a in Supporting Information S1) to the decline in aerosol‐induced heating in the middle‐ and lower troposphere over most of eastern China in 2016–2017 (Figure S11b in Supporting Information S1). This change in geopotential heights allows northerly surface winds associated with the East Asian Winter monsoon to penetrate further south, enhancing the transport of cool air from the north and west and leading to a relative cooling due to aerosols of ∼0.5 K along the southeastern coast of China between the two periods (Figure 5b). The strengthening of the East Asian Monsoon during 2016–2017 may partly account for the 25%–75% increase in dust mobilization in northwest China, as mentioned above.

The absolute magnitude of the surface PM_2.5_ enhancement due to aerosol‐radiation interactions is a function of both meteorological changes and the concentrations of PM_2.5_ absent these meteorological changes. For the simulations without aerosol‐radiation interactions over China (i.e., GEOS‐GC‐China0) surface PM_2.5_ declines between 2012–2013 and 2016‐2017 by ∼10–35 μg m^−3^ (not shown). For most of eastern China, we find that surface PM_2.5_ increases by ∼5–20 μg m^−3^ due to aerosol‐radiation interactions in 2016–2017, or about ∼5–15 μg m^−3^ less than that in 2012–2013 (Figure 5d, Figure S14c in Supporting Information S1). Reduced anthropogenic emissions and an increase in PBL heights in southeastern China account for most of this difference. Nonetheless, the relative enhancement in surface PM_2.5_ due to aerosol‐radiation impacts in 2016–2017 remains about the same at ∼10%–20%.

## 4 Discussion

Our simulations show that aerosol‐radiation interactions can significantly enhance surface PM_2.5_ concentrations by 10–40 μg m^−3^, or 10%–20% over much of China during winter. The relative enhancement is similar for both the 2012–2013 winters and the 2016–2017 winter despite declining emissions of aerosol precursors. Our findings also point to a potential role for a cloud‐snowfall‐albedo feedback in altering local and regional wintertime climate and counteracting the direct effect of reduced shortwave radiation reaching the surface due to aerosol attenuation aloft. This work also represents the first time that aerosols from the detailed chemical mechanism of GEOS‐Chem have been fully coupled to the radiation scheme in an ESM, allowing for simultaneous examination of the effect of aerosol‐radiation interactions on both climate and air pollution.

Regarding the cloud‐snowfall‐albedo feedback, we find that atmospheric warming from absorbing aerosols aloft reduces cloud liquid water content in the lower troposphere, which in turn diminishes snowfall and the surface albedo across northern China and around the Tibetan Plateau. This decline in snowfall and surface albedo leads to an unexpected surface warming of ∼0.5–1 K in central China in winter 2012–2013, counteracting the cooling due to the direct aerosol effect (i.e., reflection or absorption of shortwave radiation by aerosols aloft). Elsewhere in China, this competition between the proposed cloud‐snowfall‐albedo feedback and the direct aerosol effect leads to little surface temperature change. The heavy aerosol burden still contributes to increased stratification of the planetary boundary layer, but through warming the middle‐ and lower troposphere rather than through cooling the surface. As the aerosol burden over China declines between the winter months of 2012–2013 and 2016–2017, the cloud‐snowfall‐albedo feedback diminishes, with the net result that there is little surface temperature response to declining aerosols or even a slight cooling.

Previous studies have pointed to the role of absorbing aerosols in heating the atmosphere and stabilizing the planetary boundary layer in China (e.g., Ding et al., 2016; Miao et al., 2016; X. Wang et al., 2018). To our knowledge, however, prior studies have not detected the cloud‐snowfall‐albedo feedback which we report here. This oversight may be because these studies primarily focused on short time periods, on a scale of days to weeks (e.g., Zhang et al., 2015; Qiu et al., 2017; H. Wang et al., 2018), which likely limited the impact of aerosol‐radiation interactions on seasonal snowfall accumulation and therefore surface albedo. Inclusion of aerosol‐cloud interactions (i.e., aerosol indirect effects) in some regional studies (e.g., Qiu et al., 2017; H. Wang et al., 2018) may also have made it more difficult to detect a cloud‐snowfall‐albedo feedback. For example, aerosol‐cloud interactions could lead to additional surface cooling, which could then counteract the warming due to reductions in albedo. Other studies have reported a significant reduction in cloudiness and precipitation due to absorbing aerosols over China, but have not focused on snowfall in winter (Zhuang et al., 2013, 2019).

In many ESMs, simpler representations of chemistry than that in GEOS‐Chem may not capture the enhancement of secondary PM_2.5_ – for example, sulfate or nitrate particles – due to aerosol‐radiation interactions (e.g., Bartlett et al., 2018; Z. Li et al., 2016). The default configuration of GEOS‐ESM with GOCART aerosols is an example of such an ESM, as multiple important precursor gases are determined using prescribed fields that are not responsive to changes in meteorology or radiation (Chin et al., 2000, 2002; Colarco et al., 2010). Because secondary species tend to scatter incoming sunlight, models with such simplified chemistry may not accurately capture the balance between the cooling and warming effects of aerosols. In particular, this shortcoming may lead in turn to an underestimate of the shortwave absorption by black carbon and dust, especially during haze events over China, when scattering of incoming sunlight enhances this absorption (Bi et al., 2014). Some ESMs include treatments of the effect of aerosols on cloud condensation nuclei or the impact of black carbon deposition on snow albedo; consideration of such effects can complicate attribution of modeled changes in precipitation or snow cover (e.g., Jiang et al., 2017; Lin et al., 2018). By focusing just on aerosol‐radiation interactions, our study rules out these other pathways as causes for the simulated reductions in snow cover.

With regards to circulation changes in response to aerosol‐radiation interactions, our results agree with prior work, which show an enhancement of the East Asian winter monsoon circulation north of ∼29° and a reduction to the south of that (Liu et al., 2019). In contrast to Liu et al. (2019), which considers the impacts of aerosol on both radiation and cloud microphysics, we attribute this change solely to aerosol‐radiation interactions. For example, absorbing aerosols over the Sichuan Basin alter the latitudinal gradient in geopotential height.

Our results show the importance of considering the impacts of aerosols on regional and local climate and meteorology on seasonal timescales. Our model setup, with detailed GEOS‐Chem chemistry coupled to the radiation scheme in an ESM, allows us to identify a novel feedback involving aerosol, cloud water, snowfall and surface albedo; this feedback may be difficult to detect with other model configurations – for example, with fixed meteorological or chemical boundary conditions. Our work further shows that decreasing emissions of aerosol and aerosol precursors – as occurred between 2012–2013 and 2016–2017 – might not significantly reduce PM_2.5_ enhancement due to aerosol‐radiation interactions, contrary to what might be expected. The differences between our simulations with and without aerosol‐radiation interactions suggest ∼30%–50% of the reduction in surface PM_2.5_ seen between 2012–2013 and 2016–2017 is related to aerosol radiation‐interactions rather than a direct consequence of emissions reductions. This work implies that when designing air quality and climate policies, decision makers should consider a broad range of timescales and spatial scales in which chemical and meteorological feedbacks can occur. As absorbing aerosols play an important role in these feedbacks here by promoting cloud evaporation and stabilizing the planetary boundary layer, our work also suggests that emissions of these aerosols are an important target for policy makers and that reducing uncertainties in modeling of absorbing aerosols is an important objective for future research.

A limitation of our work is that it examines only aerosol‐radiation interactions and therefore does not encompass the full range of impacts that aerosols may have on climate and meteorology. Consideration of the effect of black carbon deposition on snow albedo would likely enhance the reductions in surface albedo that we report here. Alternatively, inclusion of aerosol‐cloud interactions, in which aerosols promote cloud formation by serving as cloud condensation nuclei, could dampen the modeled loss of cloud cover due to absorbing aerosols. Higher surface temperatures compared to MERRA‐2 for northwestern and north‐central China also suggest the possibility of excess snow melt and that modeled surface albedo may be more sensitive to minor changes in snowfall than reality. Nonetheless, using an ESM linked with detailed chemistry, such as that in GEOS‐Chem, represents an important direction for understanding the full range of impacts that aerosols can have on local and regional climate and meteorology. Many prior ESM studies have examined the one‐way impacts of aerosols on climate (e.g., Collins et al., 2017; Shindell et al., 2016), but the impact of feedbacks between aerosol and meteorology on surface PM_2.5_ concentrations has to date been less frequently considered.

## 5 Conclusion

This work represents the first time that the detailed GEOS‐Chem chemistry has been fully coupled with the radiation scheme in an ESM. Our approach allows us to examine aerosol‐radiation interactions and the subsequent effects on surface PM_2.5_ concentrations during winter months across two two‐year periods in China: 2012–2013 and 2016–2017. We propose a novel cloud‐snowfall‐albedo feedback, in which absorbing aerosols warm the middle‐ and lower troposphere, limiting the condensation of cloud liquid water and thereby reducing snowfall, snow cover, and surface albedo. This feedback counteracts the cooling effect of reduced shortwave radiation reaching the surface in the presence of reflecting and absorbing aerosols aloft. Our work shows that aerosol‐radiation interactions enhance wintertime surface PM_2.5_ over China by ∼10%–20%, mainly due to increased lower tropospheric stability and suppressed planetary boundary layer heights. The significant decline in aerosol and aerosol precursor emissions between 2012–2013 and 2016–2017 weakens the cloud‐snowfall‐albedo feedback reported here, and thus the emissions decline has little impact on reducing the relative magnitude of the effect of aerosol‐radiation interactions on surface PM_2.5_ concentrations. The important role of absorbing aerosols in our simulations suggests that black carbon may be a key target for policy makers seeking to improve air quality. Our simulations also point to the importance of policy makers considering multiple spatial and temporal scales to account for feedbacks between air quality and metrology. This work shows the potential for new insights to arise from ESMs fully coupled with detailed chemical mechanisms and for examining the impact of aerosol‐driven climate change on surface PM_2.5_.

## Supporting information

Supporting Information S1

## Data Availability

Resources supporting the model simulations were provided by the NASA Center for Climate Simulation at the Goddard Space Flight Center (https://www.nccs.nasa.gov/services/discover). Model output is available at: https://doi.org/10.7910/DVN/ZE14ST.
