# Supplementary material for: Aerosol‐Radiation Interactions in China in Winter: Competing Effects of Reduced Shortwave Radiation and Cloud‐Snowfall‐Albedo Feedbacks Under Rapidly Changing Emissions
Source: J Geophys Res Atmos. 2022 May 4;127(9):e2021JD035442. doi: 10.1029/2021JD035442 (PMC9285729; doi:10.1029/2021JD035442)
Supplement: Supplementary file 1 — Supporting Information S1 [file JGRD-127-0-s001.pdf]

AGU PUBLICATIONS

Journal of Geophysical Research – Atmospheres

Supporting Information for

Aerosol-radiation interactions in China in winter: Competing effects of reduced shortwave radiation and cloud-snowfall-albedo feedbacks under conditions of rapidly changing emissions

Jonathan M. Moch[1,2], Loretta J. Mickley[1], Christoph A. Keller[3,4], Huisheng Bian[3,4], Elizabeth W. Lundgren[1], Shixian Zhai[1], Daniel J. Jacob[1,2]

[1] John A. Paulson School of Engineering and Applied Sciences, Harvard University, Cambridge MA, USA
[2] Department of Earth and Planetary Sciences, Harvard University, Cambridge, MA, USA
[3] Global Modeling and Assimilation Office, NASA Goddard Space Flight Center, Greenbelt, MD, USA
[4] Universities Space Research Association, Columbia, MD, USA

Contents of this file

Section S1. GEOS-ESM and GOCART model details
Section S2. Model Evaluation

Figure S1. Aerosol mask used for eliminating the effect of aerosols on radiation over China
Figure S2. Comparison between GEOS-GC and MERRA-2 for surface air temperature and PBL height for DJF (2012-13 & 2016-17)
Figure S3. Comparison between GEOS-GC and GC-Offline $PM_{2.5}$ for DJF (2013 and 2016-17)
Figure S4. Impact of aerosol-radiation interactions on radiative flux and heating for 2012-13 (DJF)
Figure S5. Standard deviation of the impact of aerosol-radiation interactions on air temperature at 850 hPa for 2012-2013 (DJF) and 2016-17 (DJF)
Figure S6. Climatology and impact of aerosol-radiation interactions on selected meteorological variables for 2012-13 (DJF)
Figure S7. Mean wind speed and direction for 2012-13 (DJF)
Figure S8. Reconstructed AAOD at 500 nm from simulated mean aerosol optical depth (AOD) for December-January-February (DJF) as simulated in GEOS-GC
Figure S9. Impact of aerosol-radiation interactions at 700 hPa on short wave heating and air temperature for 2012-13 (DJF)
Figure S10. Change between 2012-13 and 2016-17 in December-January-February (DJF) in the clear sky impacts of aerosols on shortwave radiation absorbed by the atmosphere and shortwave radiation reaching the surface

Figure S11. Change in impact of aerosol-radiation interactions between 2012-13 and 2016-17 (DJF) on shortwave radiation absorbed by the atmosphere, shortwave heating rate at 850 hPa, cloud liquid water path, falling snow at the surface, and surface albedo
Figure S12. Changes between 2012-13 and 2016-17 for December-January-February (DJF) column mean single scattering albedo (SSA)
Figure S13. Impact of aerosol-radiation interaction for 2016-17 (DJF) for feedbacks involving aerosol, clouds, snow, and surface albedo
Figure S14. Effect of aerosol-radiation interactions on atmospheric stability and $PM_{2.5}$ for 2016-17 (DJF)
Figure S15. Change in impact of aerosol-radiation interactions between 2012-13 and 2016-17 (DJF) on geopotential height and windspeed at 700 hPa
Figure S16. Impact of aerosol-radiation interactions on radiative flux and heating for 2016-17 (DJF)
Figure S17. Climatology and impact of aerosol-radiation interactions on selected meteorological variables for 2016-17 (DJF)
Figure S18. Mean air temperature at 850 hPa for 2012-2013 (DJF) and 2016-17 (DJF)
Figure S19. Mean wind speed and direction for 2016-17 (DJF)
Figure S20. Impact of aerosol-radiation interactions at 700 hPa for 2016-17 (DJF) on shortwave heating and air temperature
Figure S21. Impact of aerosol-radiation interactions on atmospheric circulation patterns for 2016-2017 December-January-February (DJF)

S1: GEOS-ESM and GOCART model details

GEOS-ESM uses a modular Earth System Modeling Framework (ESMF, Hill et al., 2004). The model features a cubed sphere horizontal grid (Putman and Lin, 2007), with a hybrid sigma-pressure discretization for vertical coordinates (Simmons and Burridge, 1981). Large-scale dynamics and transport are computed with a flux-form semi-Lagrangian finite-volume dynamics scheme (Lin, 2004; Putman and Lin, 2007). Convection is parameterized with a Relaxed Arakawa-Schubert scheme (Moorthi and Suarez, 1992), and deep convection pairs this scheme with a stochastic Tokioka-type trigger function (Tokioka et al., 1988). Cloud cover and cloud water and ice volume are calculated with a single moment parametrization based on water vapor, condensate mass flux, and relative humidity, but not depending on aerosol concentration (Bacmeister et al., 2006; Rienecker et al., 2008; Molod et al., 2012). Snow cover area and albedo are derived via a three-layer snow model coupled to a catchment based hydrology model that takes into account vegetation masking by land cover type. The model yields an area-weighted composite albedo. Effects of aerosol deposition on snow are not taken into account (Koster et al., 2000; Stieglitz et al., 2001). Albedo in snow-free areas is determined using MODIS data (Mood et al., 2005; Rienecker et al., 2008). Boundary layer mixing in the model is based on Lock et al. (2000), which depends on the Richardson number (Louis et al, 1982). Over land, PBL height is calculated as a function of the bulk Richardson number (McGrath-Spangler and Molod, 2014). The radiation scheme considers absorption by aerosols in the longwave and both scattering and absorption of aerosols in the shortwave (Chou et al., 1992; Chou and Suarez, 1994).

Aerosol species in GOCART include sulfate, nitrate, ammonium, sea salt, dust, organic carbon (OC), and black carbon (BC). Sulfate is formed via gas-phase and aqueous-phase oxidation of SO_2, using prescribed oxidant fields (Chin et al., 2000; Chin et al., 2002; Colarco et al., 2010). Nitrate is divided into three size bins, while dust and sea salt each have five size bins (Bian et al., 2017; Colarco et al., 2017). Particulate nitrate formed through thermodynamic partitioning is assigned the smallest size bin, while nitrate formed from heterogeneous reactions on dust or sea salt is distributed among size bins, depending on the size of the particles on which the heterogeneous reactions occur (Bian et al., 2017; Colarco et al., 2017). BC and OC are divided into hydrophilic and hydrophobic fractions, and only primary OC is considered (Chin et al., 2002; Colarco et al., 2010).

Aerosol optical properties are calculated for each species and size bin assuming constant particle densities as well as lognormal sub-bin particle size distributions for all aerosol types other than dust. For dust, we apply a power-law distribution for the sub-bin particle number size distributions (Chin et al., 2002; Colarco et al., 2010). Calculation of aerosol optical properties accounts for the effects of relative humidity on hygroscopic growth, for most species. For dust aerosol, the optical properties are independent of relative humidity (Chin et al., 2002; Colarco et al., 2010).

S2: Model evaluation

In our model setup, GEOS-ESM is freely running and not tied to observations, except for the initialization, when one month is nudged to MERRA-2 assimilated meteorology (Section 2.4). Here we compare the 4-year DJF mean simulated surface temperature and PBL heights from GEOS-GC with those from MERRA-2. To calculate statistical significance, we conduct a pooled t-test, with one sample comprising the MERRA-2 DJF monthly means across 2012-2013 and 2016-2017 and the other sample comprising the DJF monthly means for those years across all five ensemble members. We find that GEOS-GC has a positive bias of ~4 K for much of northwestern and north-central China (Figure S1). GEOS-GC also underestimates surface air temperature compared to MERRA-2 for part of south-central China, near the eastern edge of the Tibetan Plateau. GEOS-GC underestimates PBL height compared to MERRA-2 by 25% or more for most of southern China, with the largest bias seen on the edges of the Tibetan Plateau (Figure S2). In contrast, GEOS-GC overestimates PBL height compared to MERRA-2 by ~25% for northern China along the border of Mongolia (Figure S1). These differences arise from the using the model radiation scheme with GEOS-Chem aerosols rather than GOCART aerosols and from the absence of observational data assimilation in the GEOS-GC simulations.

These differences in meteorology lead inevitably to differences in the $PM_{2.5}$ generated by GEOS-GC compared to that in GC-Offline – i.e., the offline version of GEOS-Chem driven by MERRA-2 assimilated meteorological fields. Here we compare the 2013, 2016, and 2017 ensemble mean of wintertime (DJF) surface $PM_{2.5}$ concentrations from GEOS-GC with those from the GC-Offline simulation described by Zhai et al. (2021). As the Zhai et al. (2021) simulation has very low biases in $PM_{2.5}$ compared to surface observations, the close match between GEOS-GC and GC-Offline suggests relatively small errors in the GEOS-GC $PM_{2.5}$ compared to observations To calculate statistical significance in the differences between the two sets of simulations, we conduct a pooled t-test, with monthly mean $PM_{2.5}$ concentrations from GC-Offline as one sample and monthly means of $PM_{2.5}$ across all five GEOS-GC ensemble members as the other sample. We find that the ensemble mean of our simulations has a maximum bias for $PM_{2.5}$ in DJF of ~40 $\mu g\ m^{-3}$ in a few small areas in eastern China, but these differences are generally not statistically significant across most of the region (Figure S2). Although not statistically significant, the local maxima in $PM_{2.5}$ in GEOS-GC has a larger difference compared to GC-Offline than the local maxima across much of eastern China. The higher bias over the Sichuan basin is due to the low bias in PBL heights in the region compared to MERRA-2. The PBL height bias is smaller over the east coast of China, meaning the local maxima in $PM_{2.5}$ across eastern China is less affected by this issue.

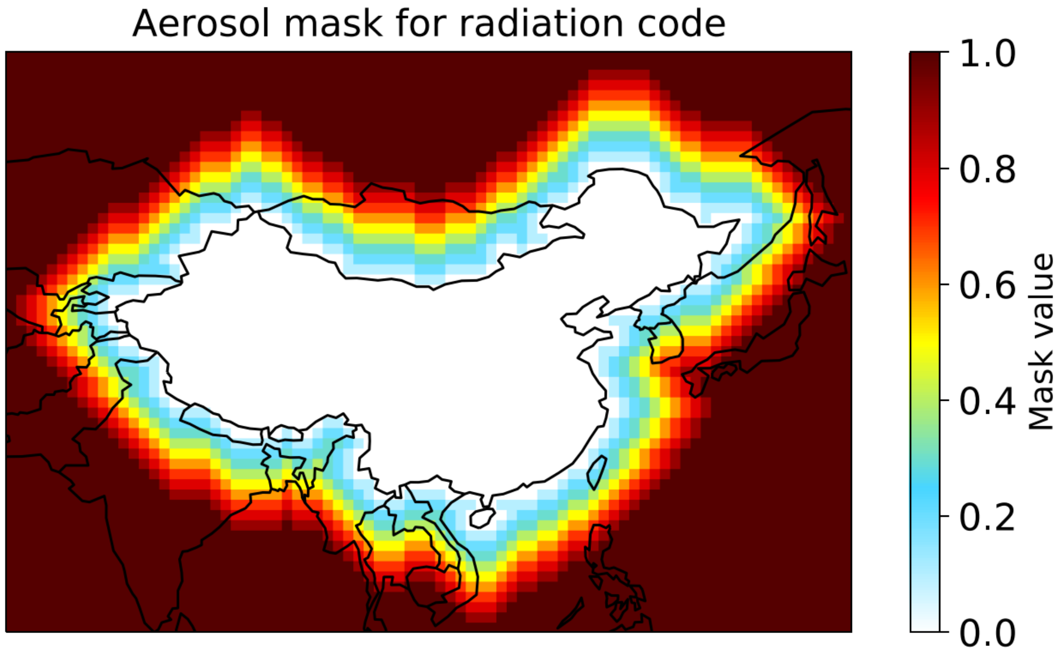

Figure S1: Aerosol mask used for eliminating the effect of aerosols on radiation over China. For the GEOS-GC-China0 simulations, aerosol mass values sent to the radiation scheme are first multiplied by the mask value in order to remove the effects of aerosol over China.

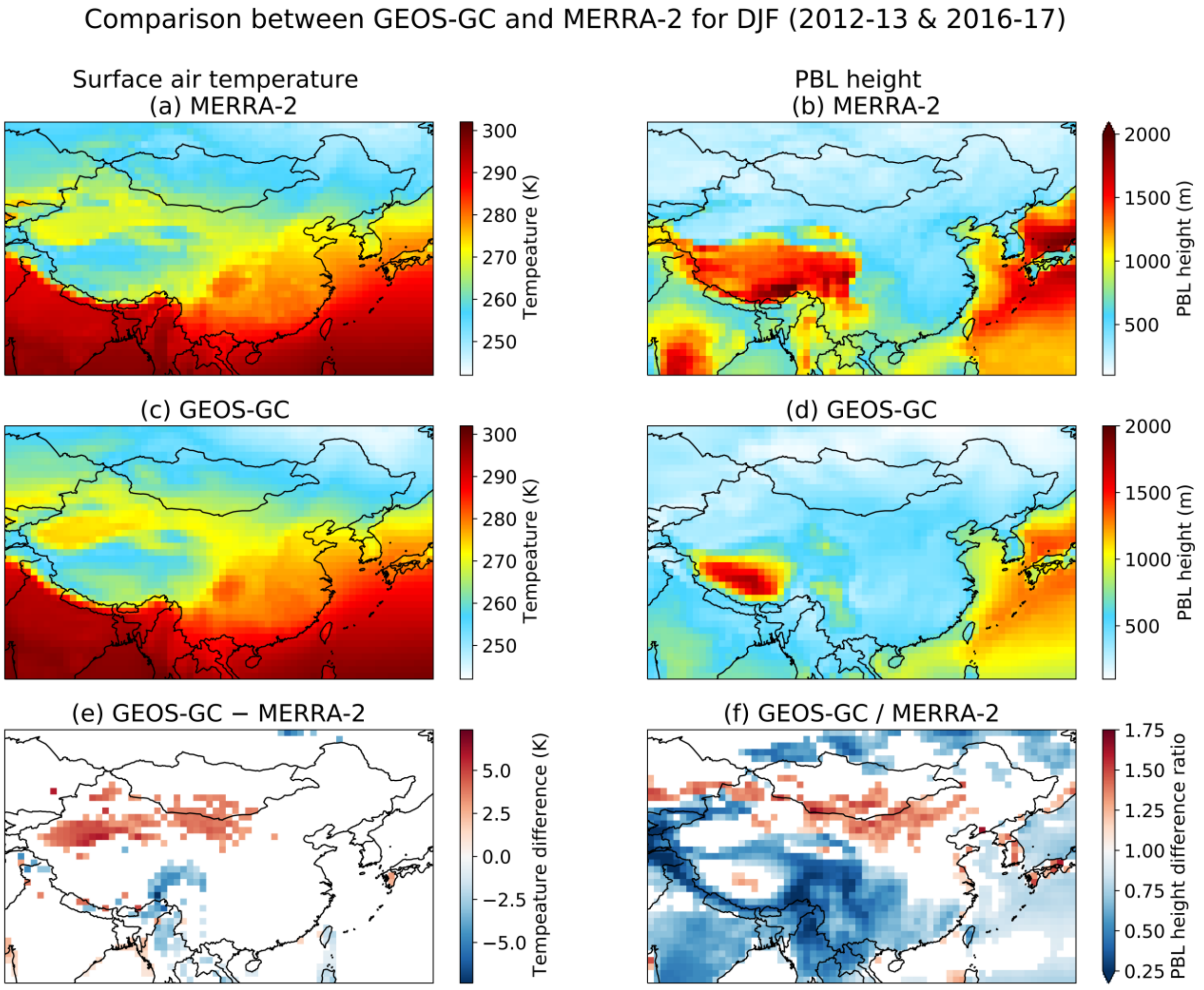

Figure S2: Comparison of mean simulated surface air temperature (left column) and PBL height (right column) in MERRA-2 (a, b) and GEOS-GC (c, d) for December-January-February (DJF) in 2012-2013 and 2016-2017. Differences between MERRA-2 and GEOS-GC are shown in panels (e) and (f), with statistically significant differences between GEOS-GC and MERRA-2 denoted as colored areas ($p < 0.05$).

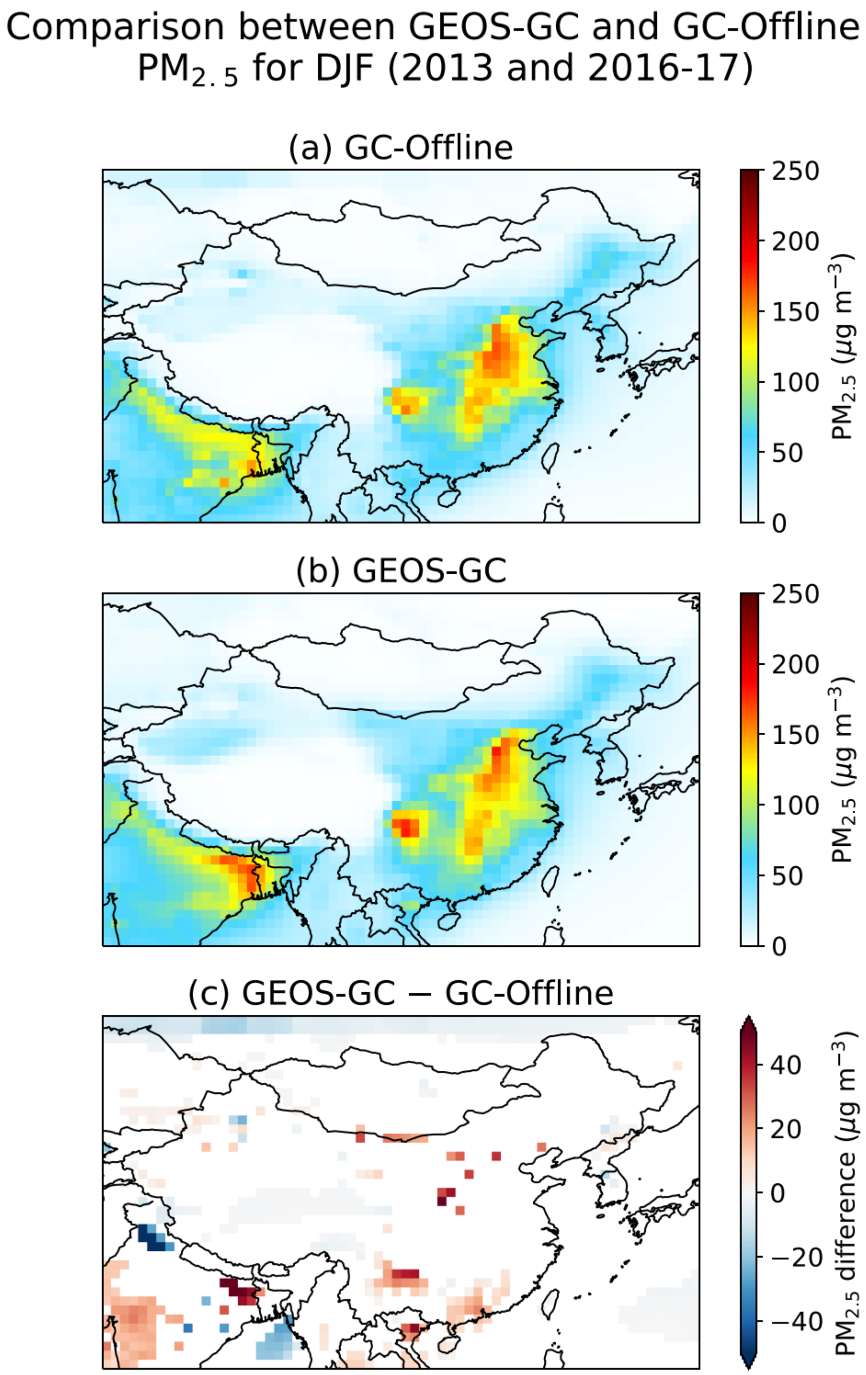

Figure S3: Comparison of mean simulated $PM_{2.5}$ concentrations at the surface in GC-Offline and GEOS-GC for December-January-February (DJF) in 2013, 2016, and 2017. Panel (a) shows results from GC-Offline (Zhai et al., 2021). Panel (b) shows results from the five-member ensemble mean of GEOS-GC run in freely running mode. Panels (c) shows the differences between GEOS-GC and GC-Offline. In panel (c) only statistically significant differences ($p < 0.05$) are shown.

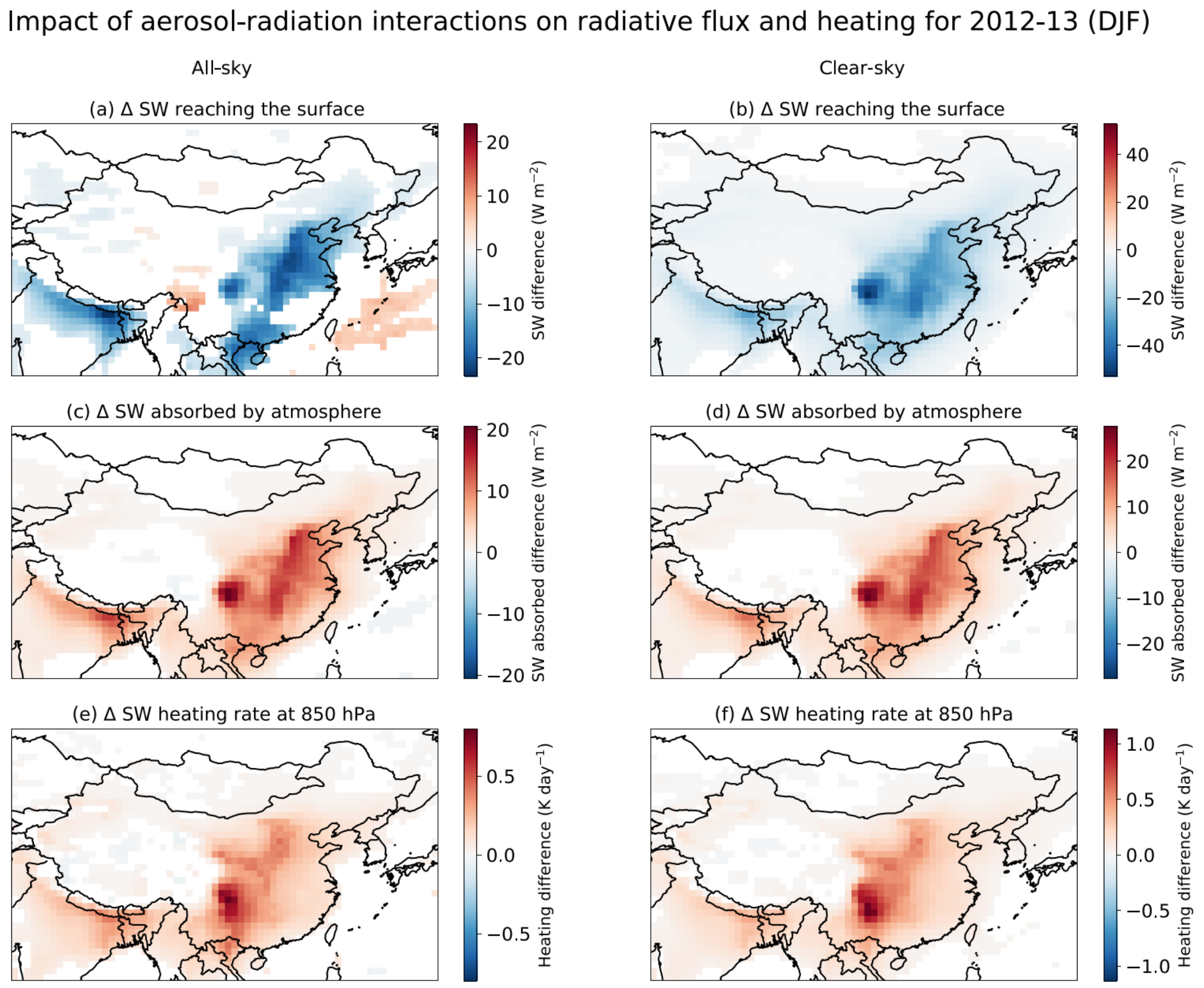

Figure S4: Impact of aerosol radiation interactions on shortwave radiative flux and heating for 2012-2013 December-January-February (DJF). Lefthand panels show results for all-sky conditions while righthand panels show clear-sky conditions. The impacts are calculated as the difference between the ensemble means for GEOS-GC and GEOS-GC-China0. Only statistically significant changes ($p < 0.05$) are shown. Panels (a) and (b) present the change in shortwave radiation reaching the surface, panels (c) and (d) the change in shortwave radiation absorbed by the entire atmospheric column, and panels (e) and (f) the change in radiative heating rate due to shortwave (SW) radiation at 850 hPa.

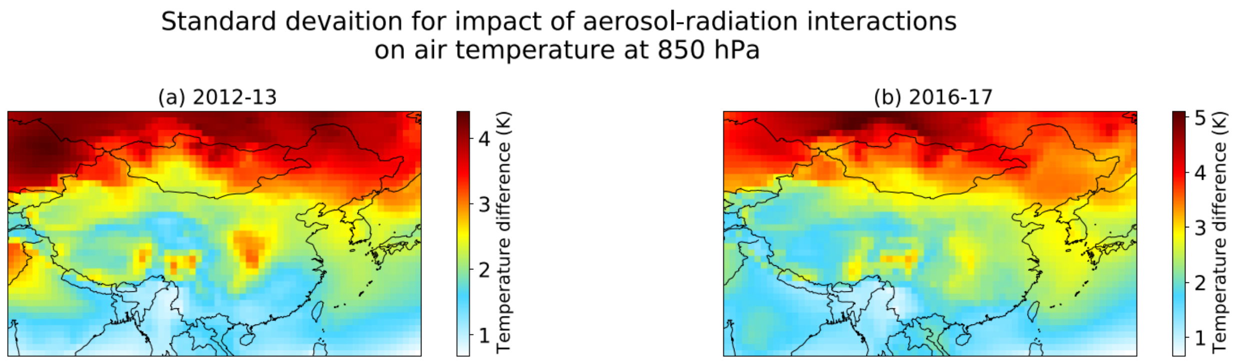

Figure S5: Standard deviation for the impact of aerosol-radiation interactions on air temperature at 850 hPa for December-January-February (DJF) in (a) 2012-13 and (b) 2016-17. The impacts are calculated as the difference between the ensemble mean for GEOS-GC and GEOS-GC-China0.

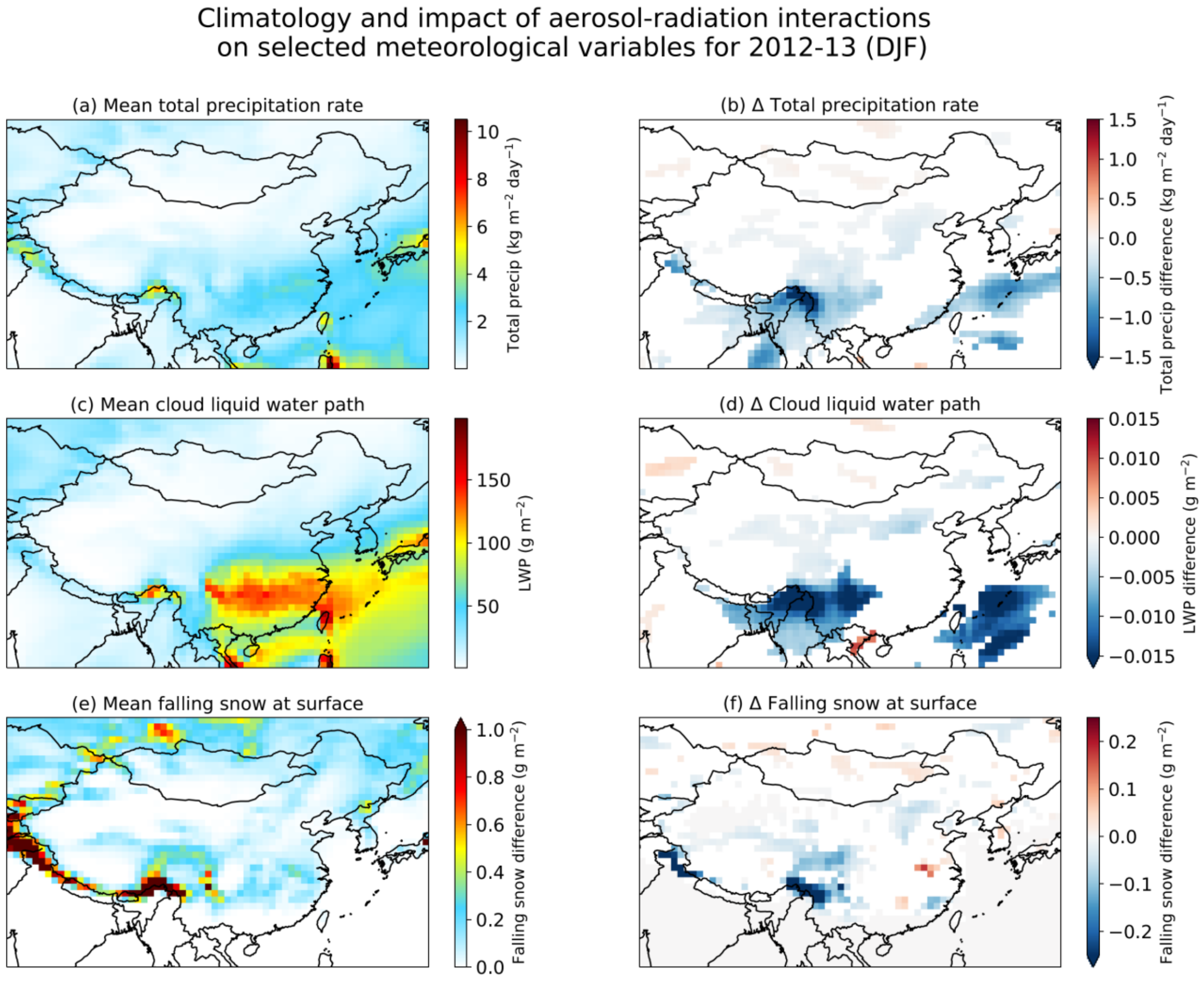

Figure S6: Impact of aerosol-radiation interactions for 2012-2013 in December-January-February (DJF) for selected variables. The left column shows the mean GEOS-GC values of (a) total precipitation rate and (c) cloud liquid water path, and (e) snowfall at the surface. The right column shows the impacts of aerosol-radiation interactions on (b) precipitation rate, (d) cloud liquid water path, and (f) snowfall at the surface. The impacts are shown as difference between the ensemble mean for GEOS-GC and GEOS-GC-China0. Colored areas indicate those regions where differences are statistically significant changes ($p < 0.05$).

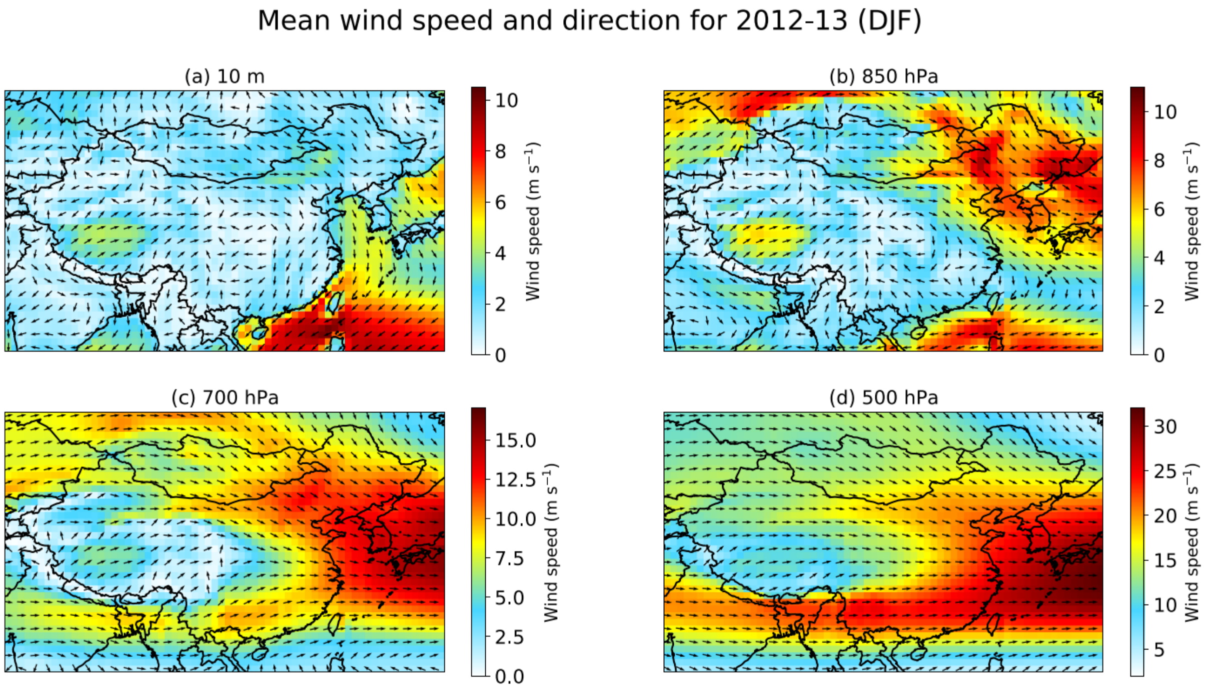

Figure S7: Mean wind speed and wind direction for 2012-2013 in December-January-February (DJF) at (a) 10-m elevation, (b) 850 hPa, (c) 700 hPa, and (d) 500 hPa, as simulated in GEOS-GC.

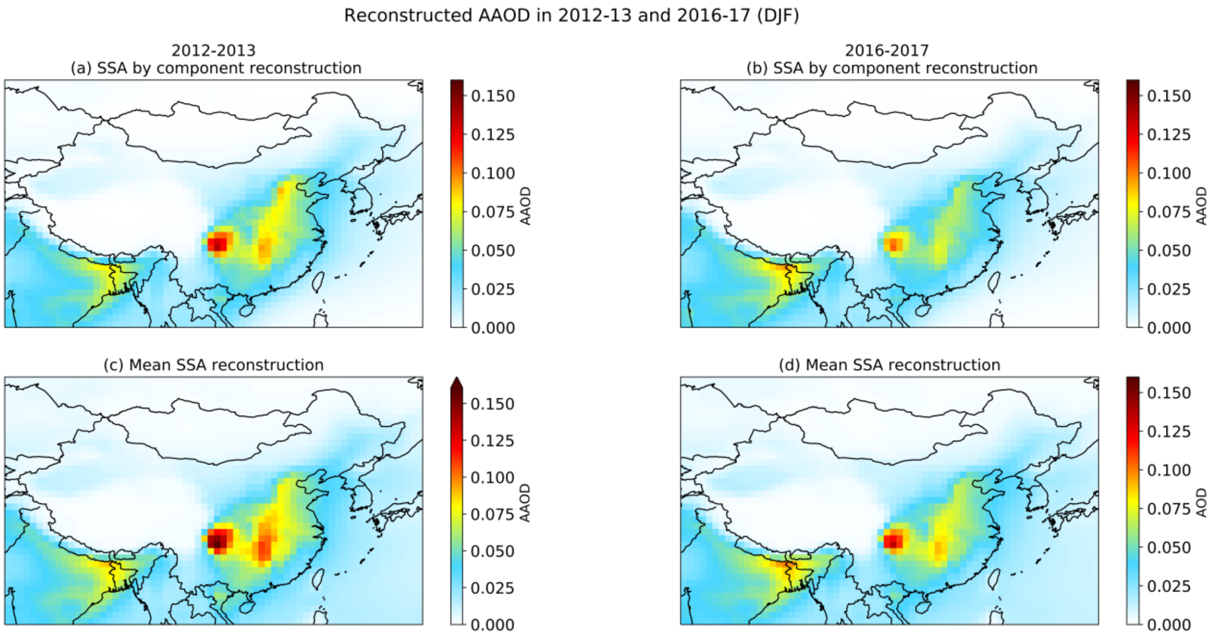

Figure S8: Reconstructed AAOD at 550 nm from simulated mean aerosol optical depth (AOD) for December-January-February (DJF) as simulated in GEOS-GC for (a,c) 2012-13 and (b,d) 2016-17. Panels (a) and (b) use an assumed single scattering albedo (SSA) of 0 for black carbon, 0.93 for dust (Colarco et al., 2010), and 0.9 for organic aerosol and brown carbon. Panels (c) and (d) use an assumed mean SSA of 0.88 for East Asian aerosol, derived from AERONET retrievals of mixed East Asian aerosol (Li et al., 2015).

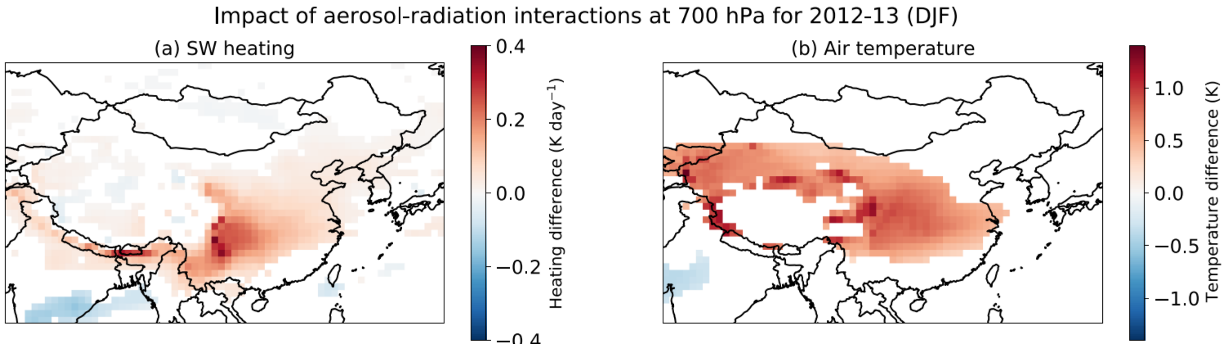

Figure S9: Impact of aerosol radiation interactions at 700 hPa on (a) all-sky shortwave radiative heating and (b) air temperature for 2012-2013 December-January-February (DJF). The impacts are calculated as the difference between the ensemble means for GEOS-GC and GEOS-GC-China0. Only statistically significant changes ($p < 0.05$) are shown.

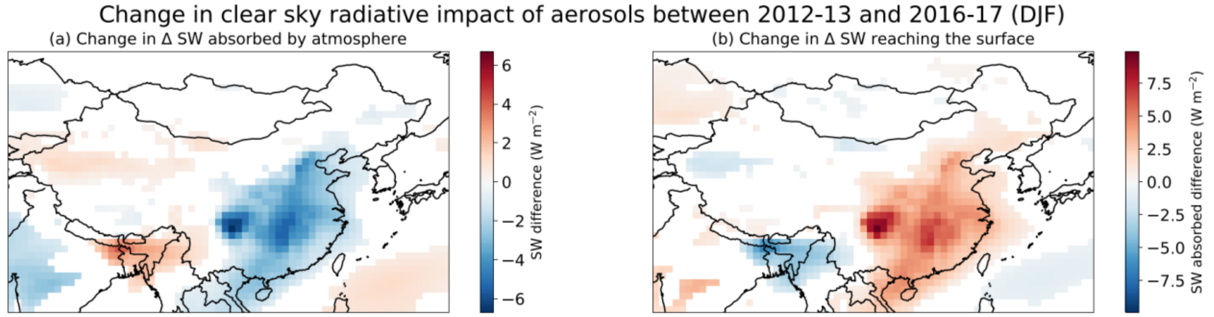

Figure S10: Change between 2012-13 and 2016-17 in December-January-February (DJF) in the clear sky impacts of aerosols on (a) shortwave radiation absorbed by the atmosphere and (b) shortwave radiation reaching the surface. The impacts are calculated as the difference between the ensemble mean for GEOS-GC and GEOS-GC-China0. Only statistically significant changes ($p < 0.05$) are shown.

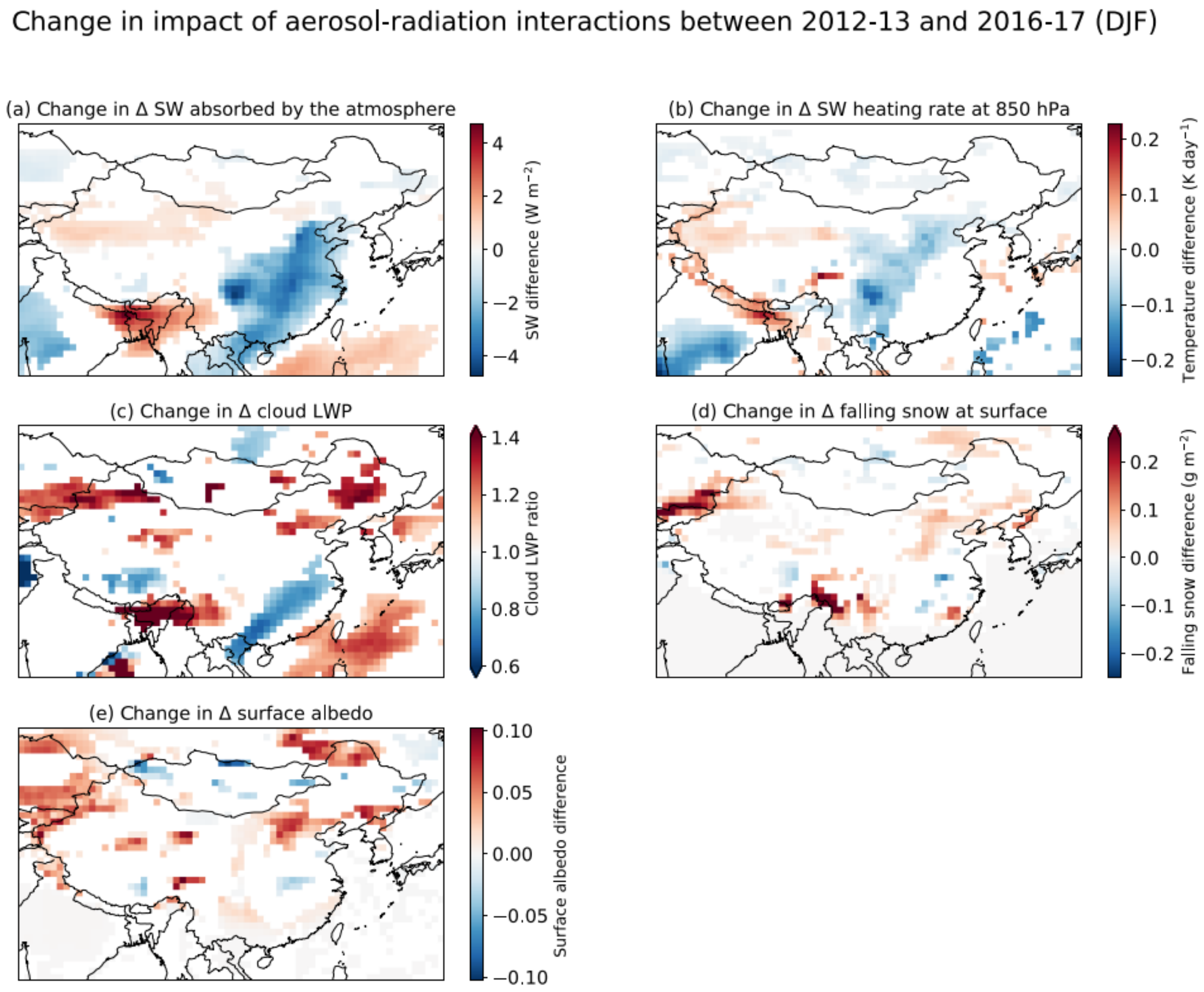

Figure S11: Changes between 2012-13 and 2016-17 for December-January-February (DJF) in the impact of aerosol radiation interactions on (a) shortwave radiation absorbed by the atmosphere, (b) shortwave heating rate for all-sky conditions, (c) cloud liquid water path, (d) snowfall at the surface, and (e) surface albedo. The panels show the differences between 2016-17 and 2012-13 in the difference of the ensemble mean for GEOS-GC and GEOS-GC-China0, except for panel (c), which shows the ratio. Only statistically significant changes ($p < 0.05$) are shown.

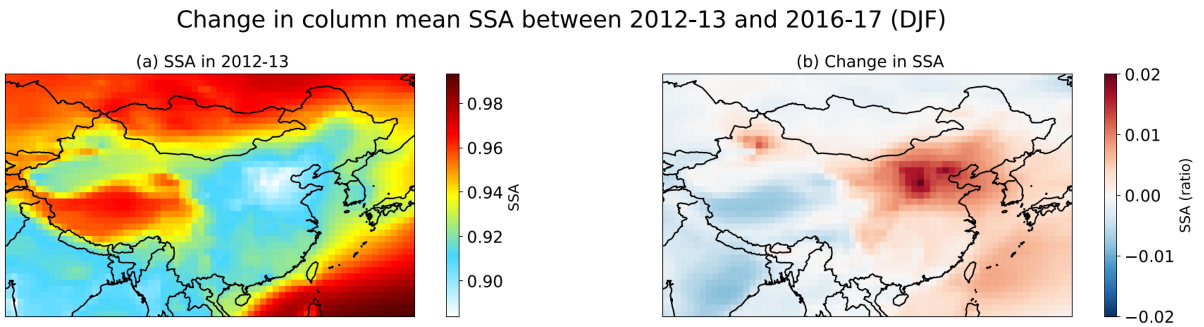

Figure S12: (a) Single scattering albedo (SSA) in 2012-2013 and (b) the change in SSA between 2012-13 and 2016-17. Both panels show the column mean, averaged across the GEOS-GC ensembles, for December-January-February (DJF). AAOD is reconstructed at 550 nm from simulated mean aerosol optical depth (AOD) for December-January-February (DJF), assuming an SSA of 0 for black carbon, 0.93 for dust (Colarco et al., 2010), and 0.9 for organic aerosol and brown carbon.

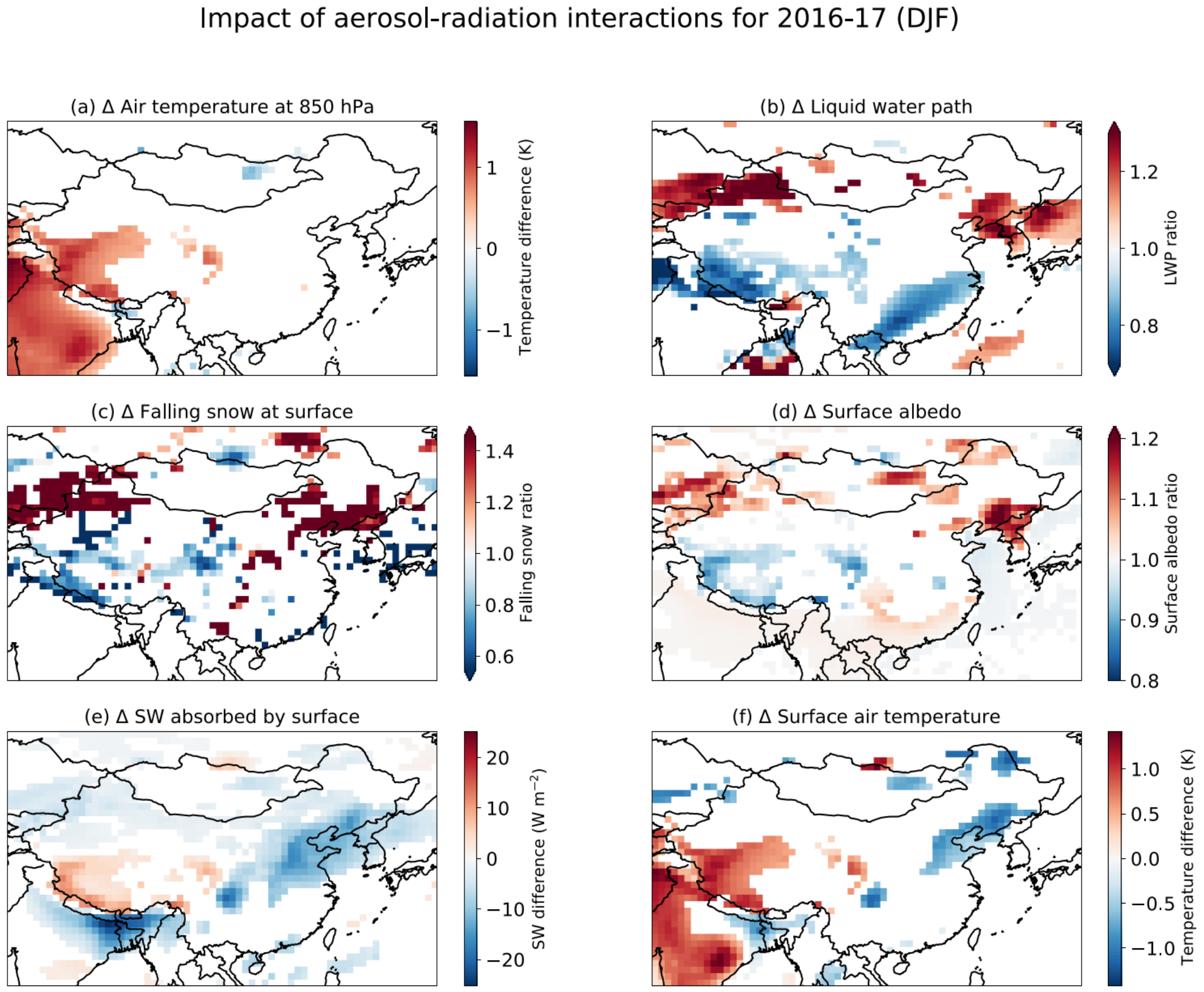

Figure S13: Feedbacks involving aerosol, clouds, snow, and surface albedo. Panels show the impacts of aerosol-radiation interactions during 2016-2017 winters (DJF) over China on (a) air temperature at 850 hPa, (b) liquid water path (LWP), (c) falling snow at the surface, (d) surface albedo, (e) shortwave radiation (SW) absorbed by the surface, and (f) surface air temperature. Panels (a), (e), and (f) show the difference between the ensemble mean for GEOS-GC and GEOS-GC-China0, while panels (b), (c), and (d) show the ratio. Colored areas indicate those regions where differences are statistically significant ($p < 0.05$).

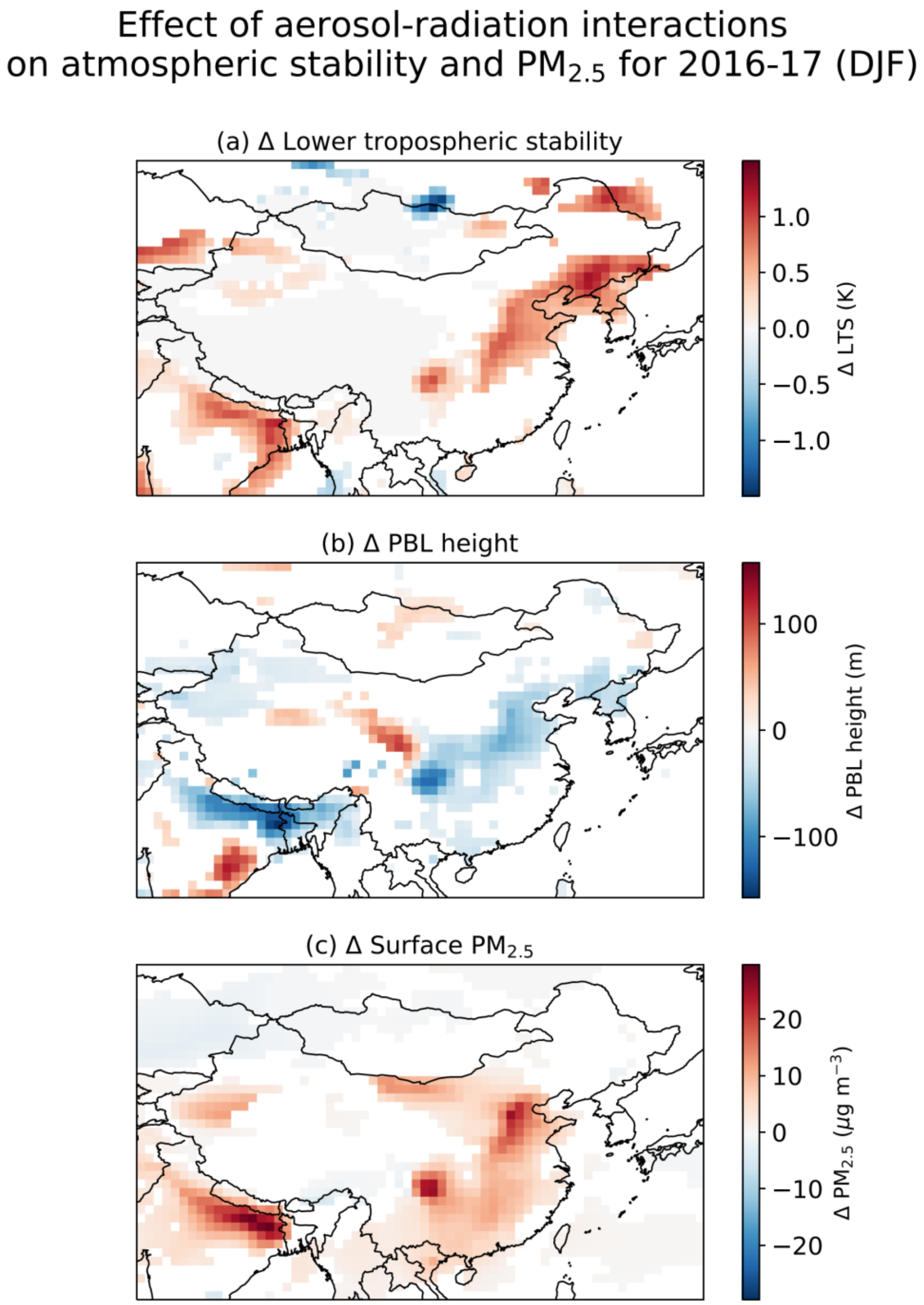

Figure S14: Impact of aerosol-radiation interactions on atmospheric stability and surface $PM_{2.5}$ concentrations. Panel (a) shows the change in lower tropospheric stability, defined as the difference in potential temperature between 850 and 1000 hPa, due to aerosol-radiation interactions; panel (b), the change in planetary boundary layer height; and panel (c), the change in surface $PM_{2.5}$. The impacts are shown as difference between the ensemble mean for GEOS-GC and GEOS-GC-China0. Colored areas indicate those regions where differences are statistically significant changes ($p < 0.05$).

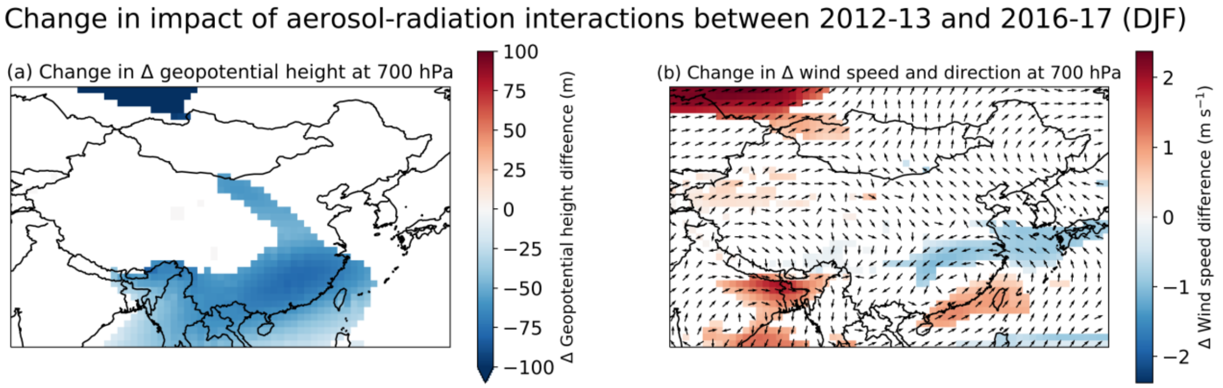

Figure S15: Change between 2012-13 and 2016-17 in December-January-February (DJF) in the impacts of aerosol-radiation interactions on (a) geopotential height at 700 hPa and (b) windspeed and direction at 700 hPa. The impacts are calculated as the difference between the ensemble mean for GEOS-GC and GEOS-GC-China0. Only statistically significant changes ($p < 0.05$) are shown for geopotential height and wind speed. Arrows in panel (b) show the net direction of the wind changes. Blue-shaded regions thus indicate a decrease in net wind speed in the opposite direction of the overlying arrows.

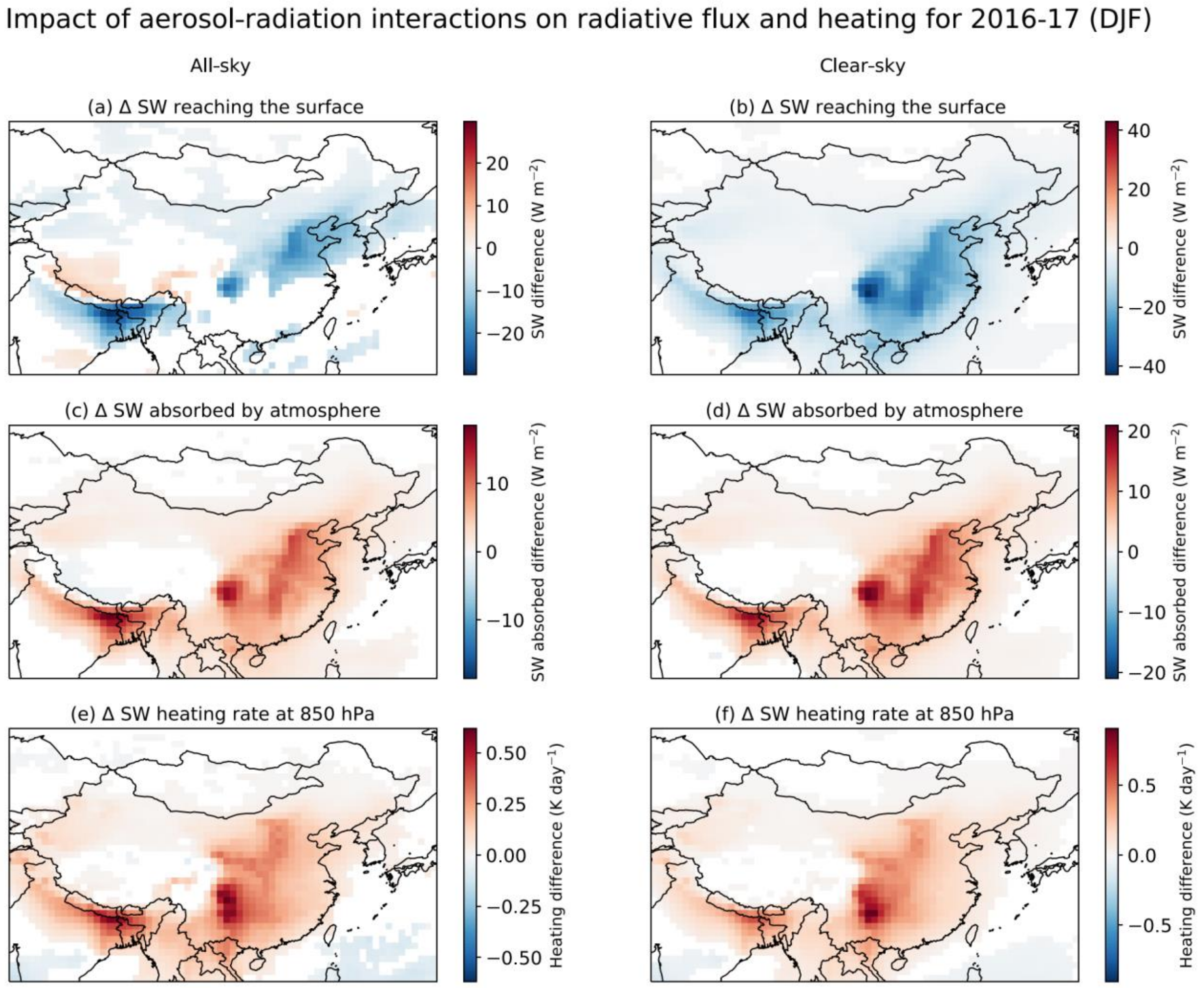

Figure S16: Impact of aerosol radiation interactions on shortwave radiative flux and heating for 2016-2017 December-January-February (DJF). Lefthand panels show results for all-sky conditions while righthand panels show clear-sky conditions. The impacts are calculated as the difference between the ensemble means for GEOS-GC and GEOS-GC-China0. Only statistically significant changes ($p < 0.05$) are shown. Panels (a) and (b) present the change in shortwave radiation reaching the surface, panels (c) and (d) the change in shortwave radiation absorbed by the entire atmospheric column, and panels (e) and (f) the change in radiative heating rate due to shortwave (SW) radiation at 850 hPa.

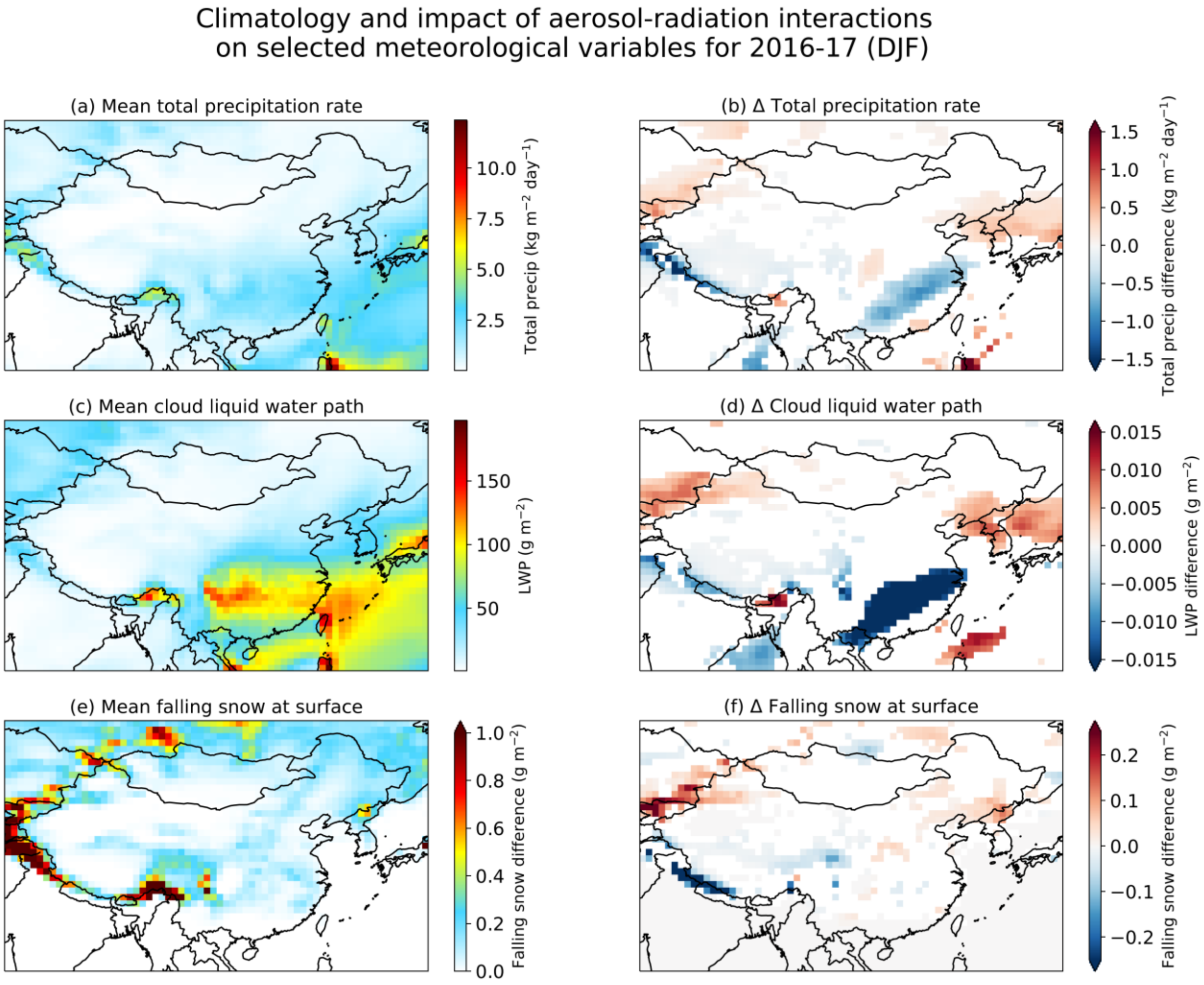

Figure S17: Impact of aerosol-radiation interactions for 2016-2017 December-January-February (DJF) for selected variables. The left column shows the mean GEOS-GC values of (a) total precipitation rate and (c) cloud liquid water path, and (e) snowfall at the surface. The right column shows the impacts of aerosol-radiation interactions on (b) precipitation rate, (d) cloud liquid water path, and (f) snowfall at the surface. Only statistically significant changes ($p < 0.05$) are shown.

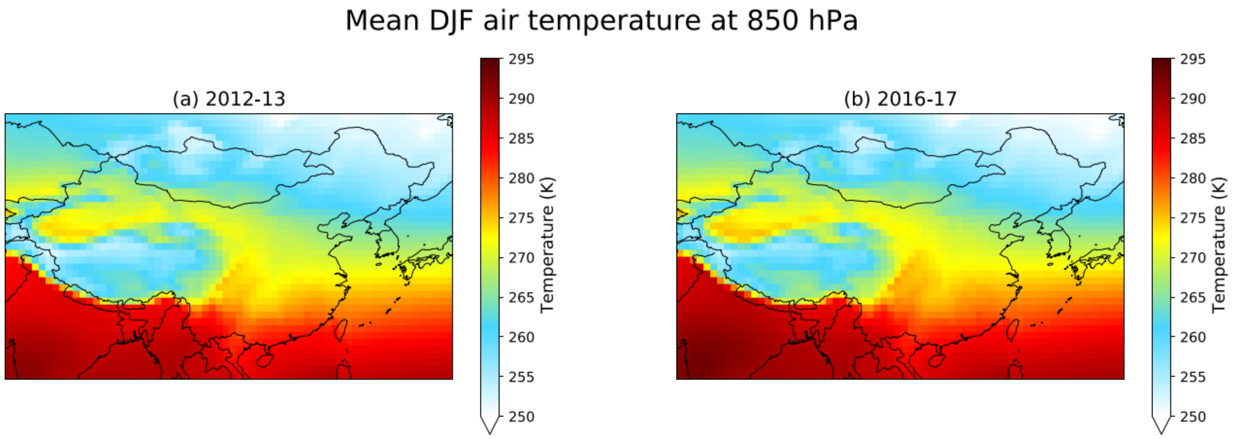

Figure S18: Mean December-January-February (DJF) air temperature at 850 hPa for (a) 2012-2013 and (b) 2016-2017.

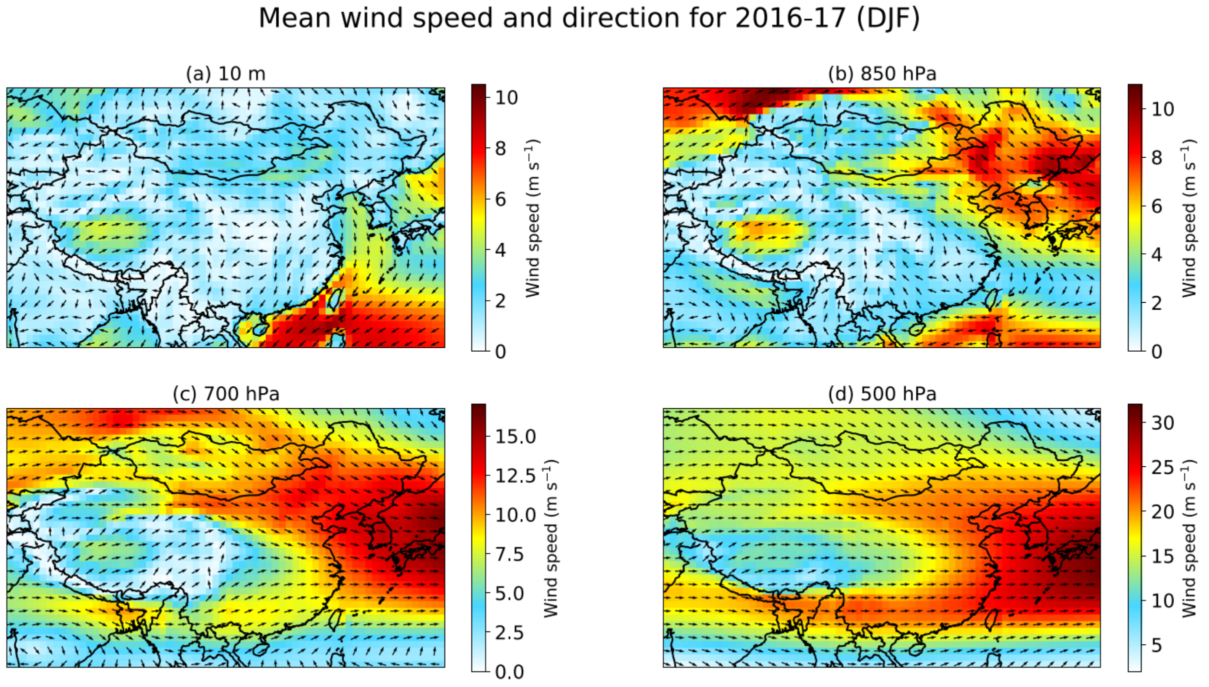

Figure S19: Mean wind speed and wind direction for 2016-2017 December-January-February (DJF) at (a) 10 m elevation, (b) 850 hPa, (c) 700 hPa, and (d) 500 hPa, as simulated in GEOS-GC.

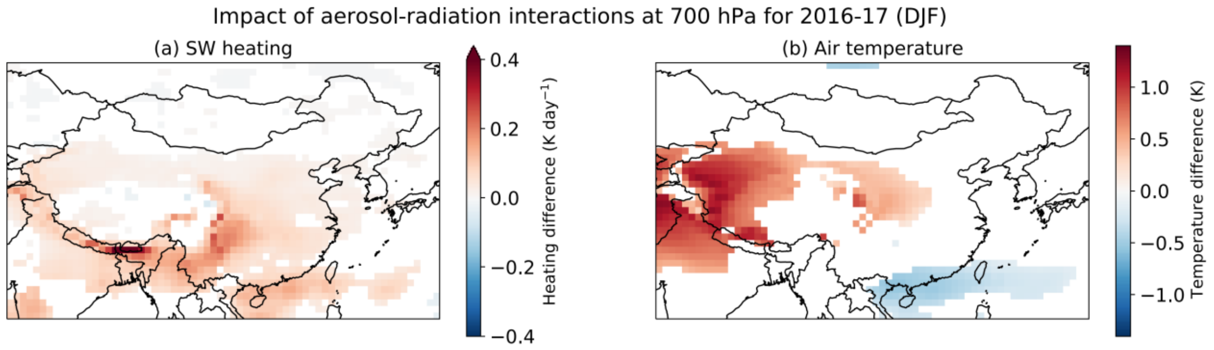

Figure S120: Impact of aerosol radiation interactions at 700 hPa on (a) all-sky shortwave radiative heating and (b) air temperature for 2016-2017 December-January-February (DJF). The impacts are calculated as the difference between the ensemble means for GEOS-GC and GEOS-GC-China0. Only statistically significant changes ($p < 0.05$) are shown.

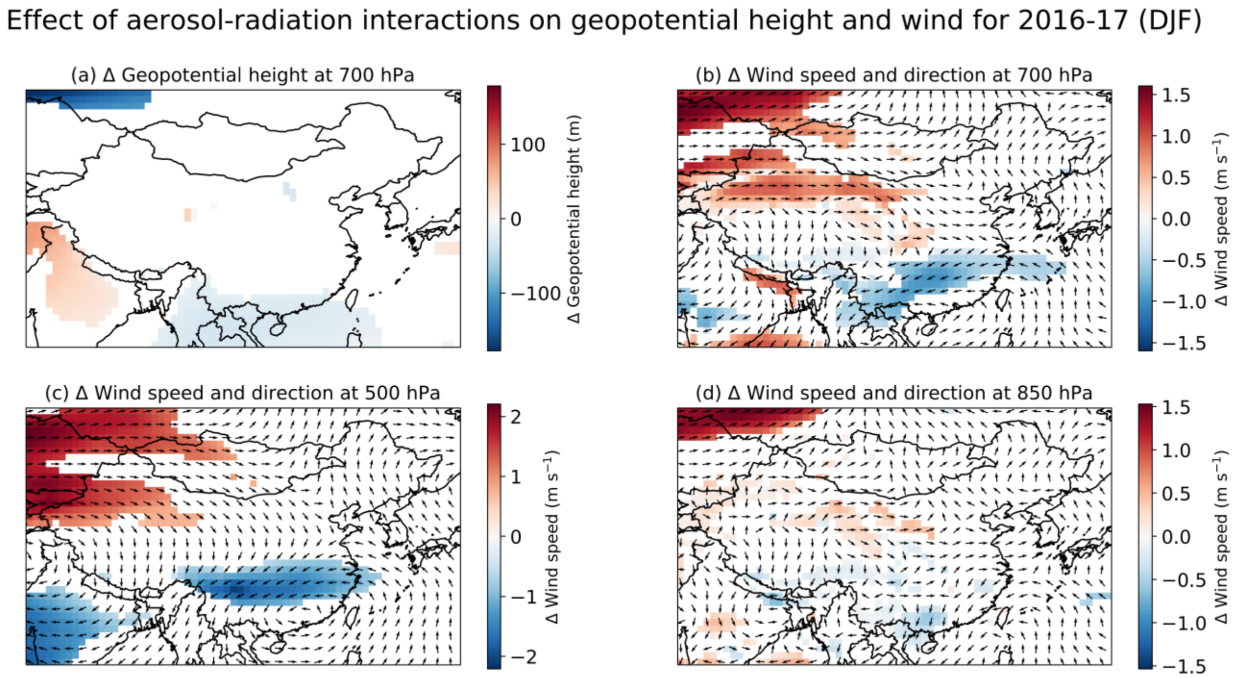

Figure S21: Impact of aerosol-radiation interactions on atmospheric circulation patterns for 2016-2017 December-January-February (DJF). The panels show the changes in geopotential height (a) and in wind speed and direction at (b) 700 hPa, (c) 500 hPa, and (d) 850 hPa. The impacts are calculated as the difference between the ensemble mean for GEOS-GC and GEOS-GC-China0. Only statistically significant changes ($p<0.05$) are shown for geopotential height and wind speed. Arrows for (b-c) show the net direction of the wind changes. Blue-shaded regions thus indicate a decrease in wind speed in the opposite direction of the overlying arrows.

References:

Bacmeister, J. T., Suarez, M. J., & Robertson, F. R. (2006). Rain Reevaporation, Boundary Layer–Convection Interactions, and Pacific Rainfall Patterns in an AGCM. *Journal of the Atmospheric Sciences*, *63*(12), 3383–3403. https://doi.org/10.1175/JAS3791.1

Bian, H., Chin, M., Hauglustaine, D. A., Schulz, M., Myhre, G., Bauer, S. E., et al. (2017). Investigation of global particulate nitrate from the AeroCom phase III experiment. *Atmospheric Chemistry and Physics*, *17*(21), 12911–12940. https://doi.org/10.5194/acp-17-12911-2017

Chou, M.-D. (1992). A Solar Radiation Model for Use in Climate Studies. *Journal of the Atmospheric Sciences*, *49*(9), 762–772. https://doi.org/10.1175/1520-0469(1992)049<0762:ASRMFU>2.0.CO;2

Chou, M.-D., & Suarez, M. J. (1994). *An efficient thermal infrared radiation parameterization for use in general circulation models* (NASA Tech. Memorandum No. NASA/TM-1994–104606). Goddard Space Flight Center Greenbelt, Maryland 20771: National Aeronautics and Space Administration.

Colarco, P. S. (2017, June). *Update on the NASA GEOS-5 Aerosol Forecasting and Data Assimilation System*. Retrieved from https://ntrs.nasa.gov/search.jsp?R=20170006600

Darmenov, A., & da Silva, A. M. (2015). *The Quick Fire Emissions Dataset (QFED): Documentation of versions 2.1, 2.2 and 2.4* (Technical Report Series on Global Modeling and Data Assimilation, Volume 38 No. NASA/TM–2015–104606). Goddard Space Flight Center Greenbelt, Maryland 20771: National Aeronautics and Space Administration. Retrieved from https://gmao.gsfc.nasa.gov/pubs/docs/Darmenov796.pdf

Ginoux, P., Chin, M., Tegen, I., Prospero, J. M., Holben, B., Dubovik, O., & Lin, S.-J. (2001). Sources and distributions of dust aerosols simulated with the GOCART model. *Journal of Geophysical Research: Atmospheres*, *106*(D17), 20255–20273. https://doi.org/10.1029/2000JD000053

Gong, S. L. (2003). A parameterization of sea-salt aerosol source function for sub- and super-micron particles. *Global Biogeochemical Cycles*, *17*(4). https://doi.org/10.1029/2003GB002079

Hill, C., DeLuca, C., Balaji, Suarez, M., & Silva, A. D. (2004). The architecture of the Earth System Modeling Framework. *Computing in Science Engineering*, *6*(1), 18–28. https://doi.org/10.1109/MCISE.2004.1255817

Janssens-Maenhout, G., Crippa, M., Guizzardi, D., Dentener, F., Muntean, M., Pouliot, G., et al. (2015). HTAP_v2.2: a mosaic of regional and global emission grid maps for 2008 and 2010 to study hemispheric transport of air pollution. *Atmospheric Chemistry and Physics*, *15*(19), 11411–11432. https://doi.org/10.5194/acp-15-11411-2015

Koster, R. D., Suarez, M. J., Ducharne, A., Stieglitz, M., & Kumar, P. (2000). A catchment-based approach to modeling land surface processes in a general circulation model: 1. Model structure. *Journal of Geophysical Research: Atmospheres*, *105*(D20), 24809–24822. https://doi.org/10.1029/2000JD900327

Li, J., Carlson, B. E., & Lacis, A. A. (2015). Using single-scattering albedo spectral curvature to characterize East Asian aerosol mixtures. Journal of Geophysical Research: Atmospheres, 120(5), 2037–2052. https://doi.org/10.1002/2014JD022433

Lin, S.-J. (2004). A "Vertically Lagrangian" Finite-Volume Dynamical Core for Global Models. *Monthly Weather Review*, *132*(10), 2293–2307. https://doi.org/10.1175/1520-0493(2004)132<2293:AVLFDC>2.0.CO;2

Lock, A. P., Brown, A. R., Bush, M. R., Martin, G. M., & Smith, R. N. B. (2000). A New Boundary Layer Mixing Scheme. Part I: Scheme Description and Single-Column Model Tests. *Monthly Weather Review*, *128*(9), 3187–3199. https://doi.org/10.1175/1520-0493(2000)128<3187:ANBLMS>2.0.CO;2

Louis, J.-F., Tiedtke, M., & Geleyn, J.-F. (1982). A short history of the PBL parameterization at ECMWF. In *Workshop on Planetary Boundary Layer parameterization, 25-27 November 1981* (pp. 59–79). Shinfield Park, Reading: ECMWF. Retrieved from https://www.ecmwf.int/node/10845

McGrath-Spangler, E. L., & Molod, A. (2014). Comparison of GEOS-5 AGCM planetary boundary layer depths computed with various definitions. *Atmospheric Chemistry and Physics, 14*(13), 6717–6727. https://doi.org/10.5194/acp-14-6717-2014

Molod, A., Takacs, L., Suárez, M. J., Bacmeister, J., Song, I.-S., & Eichmann, A. (2012). *The GEOS-5 Atmospheric General Circulation Model: Mean Climate and Development from MERRA to Fortuna* (Technical Report Series on Global Modeling and Data Assimilation, Volume 28 No. NASA/TM–2012-104606). Goddard Space Flight Center Greenbelt, Maryland 20771: National Aeronautics and Space Administration. Retrieved from https://gmao.gsfc.nasa.gov/pubs/docs/tm28.pdf

Moody, E. G., King, M. D., Platnick, S., Schaaf, C. B., & Gao, F. (2005). Spatially complete global spectral surface albedos: value-added datasets derived from Terra MODIS land products. *IEEE Transactions on Geoscience and Remote Sensing*, *43*(1), 144–158. https://doi.org/10.1109/TGRS.2004.838359

Moorthi, S., & Suarez, M. J. (1992). Relaxed Arakawa-Schubert. A Parameterization of Moist Convection for General Circulation Models. *Monthly Weather Review*, *120*(6), 978–1002. https://doi.org/10.1175/1520-0493(1992)120<0978:RASAPO>2.0.CO;2

Orbe, C., Oman, L. D., Strahan, S. E., Waugh, D. W., Pawson, S., Takacs, L. L., & Molod, A. M. (2017). Large-Scale Atmospheric Transport in GEOS Replay Simulations. *Journal of Advances in Modeling Earth Systems*, *9*(7), 2545–2560. https://doi.org/10.1002/2017MS001053

Putman, W. M., & Lin, S.-J. (2007). Finite-volume transport on various cubed-sphere grids. *Journal of Computational Physics*, *227*(1), 55–78. https://doi.org/10.1016/j.jcp.2007.07.022

Rienecker, M., Suarez, M. J., Todling, R., Bacmeister, J., Takacs, L., Liu, H.-C., et al. (2008). *The GEOS-5 Data Assimilation System—Documentation of Versions 5.0.1, 5.1.0, and 5.2.0* (Technical Report Series on Global Modeling and Data Assimilation, Volume 27 No. NASA/TM–2008–104606). Goddard Space Flight Center Greenbelt, Maryland 20771: National Aeronautics and Space Administration. Retrieved from https://gmao.gsfc.nasa.gov/pubs/docs/tm27.pdf

Simmons, A. J., & Burridge, D. M. (1981). An Energy and Angular-Momentum Conserving Vertical Finite-Difference Scheme and Hybrid Vertical Coordinates. *Monthly Weather Review*, *109*(4), 758–766. https://doi.org/10.1175/1520-0493(1981)109<0758:AEAAMC>2.0.CO;2

Stieglitz, M., Ducharne, A., Koster, R., & Suarez, M. (2001). The Impact of Detailed Snow Physics on the Simulation of Snow Cover and Subsurface Thermodynamics at Continental Scales. *Journal of Hydrometeorology*, *2*(3), 228–242. https://doi.org/10.1175/1525-7541(2001)002<0228:TIODSP>2.0.CO;2

TOKIOKA, T., YAMAZAKI, K., KITOH, A., & OSE, T. (1988). The equatorial 30-60 day oscillation and the Arakawa-Schubert penetrative cumulus parameterization. *The Equatorial 30-60 Day Oscillation and the Arakawa-Schubert Penetrative Cumulus Parameterization*, *66*(6), 883–901.

Zhai, S., Jacob, D. J., Wang, X., Liu, Z., Wen, T., Shah, V., et al. (2021). Control of particulate nitrate air pollution in China. *Nature Geoscience*, *14*(6), 389–395. https://doi.org/10.1038/s41561-021-00726-z
